# Thresholding of cryo-EM density maps by false discovery rate control

**DOI:** 10.1107/S2052252518014434

**Published:** 2019-01-01

**Authors:** Maximilian Beckers, Arjen J. Jakobi, Carsten Sachse

**Affiliations:** a Structural and Computational Biology Unit, European Molecular Biology Laboratory (EMBL), Meyerhofstrasse 1, 69117 Heidelberg, Germany; b Faculty of Biosciences, European Molecular Biology Laboratory (EMBL), Meyerhofstrasse 1, 69117 Heidelberg, Germany; c Hamburg Unit c/o DESY, Notkestrasse 85, 22607 Hamburg, Germany; d The Hamburg Centre for Ultrafast Imaging (CUI), Luruper Chaussee 149, 22761 Hamburg, Germany; e Ernst Ruska-Centre for Microscopy and Spectroscopy with Electrons (ER-C-3/Structural Biology), Forschungszentrum Jülich, 52425 Jülich, Germany

**Keywords:** electron cryo-microscopy, signal detection, false discovery rate, cryo-EM density, subtomogram averaging, local resolution, ligand binding

## Abstract

Cryo-EM now commonly generates close-to-atomic resolution as well as intermediate resolution maps from macromolecules observed in isolation and *in situ*. Interpreting these maps remains a challenging task owing to poor signal in the highest resolution shells and the necessity to select a threshold for density analysis. In order to facilitate this process, a statistical framework for the generation of confidence maps by multiple hypothesis testing and false discovery rate (FDR) control has been developed. In this way, three-dimensional confidence maps contain signal separated from background noise in the form of local detection rates of EM density values. It is demonstrated that confidence maps and FDR-based thresholding can be used for the interpretation of near-atomic resolution single-particle structures as well as lower resolution maps determined by subtomogram averaging. Confidence maps represent a conservative way of interpreting molecular structures owing to minimized noise. At the same time they provide a detection error with respect to background noise, which is associated with the density and is particularly beneficial for the interpretation of weaker cryo-EM densities in cases of conformational flexibility and lower occupancy of bound molecules and ions in the structure.

## Abstract 2

The interpretation of cryo-EM density by atomic models remains a daunting task owing to the identification of appropriate threshold levels at low signal-to-noise ratios in high-resolution shells and weak structural features. It is shown that transforming cryo-EM densities to confidence maps followed by false discovery rate-based thresholding provides a robust way to interpret cryo-EM structures.

## 1. Introduction   

Cryo-EM-based structure determination has undergone remarkable technological advances over the past few years, leading to a sudden multiplication of near-atomic resolution structures (Patwardhan, 2017 ▸). Before these transformative changes, only highly regular specimens such as helical or icosahedral viruses could be resolved in such detail (Unwin, 2005 ▸; Sachse *et al.*, 2007 ▸; Zhang *et al.*, 2008 ▸; Yonekura *et al.*, 2003 ▸; Yu *et al.*, 2008 ▸; Ge & Zhou, 2011 ▸). With the advent of direct electron detectors (McMullan *et al.*, 2016 ▸) and simultaneous improvements in image-processing software (Scheres, 2012*b* ▸; Lyumkis *et al.*, 2013 ▸; Punjani *et al.*, 2017 ▸), smaller, less regular and more heterogeneous single-particle specimens became amenable to routine imaging below 4 Å resolution (Bai *et al.*, 2013 ▸; Li *et al.*, 2013 ▸; Liao *et al.*, 2013 ▸). Recently, the highest resolution structures have become available at ∼2 Å resolution (Merk *et al.*, 2016 ▸; Bartesaghi *et al.*, 2018 ▸, 2015 ▸) and sub-4 Å resolution structures of molecules below 100 kDa have been resolved from images obtained with and without an optical phase plate (Merk *et al.*, 2016 ▸; Khoshouei *et al.*, 2017 ▸). These studies established technical routines for the determination of atomic models of structures that it was previously thought to be impossible to resolve by cryo-EM or any other technique (Bai *et al.*, 2015 ▸; Galej *et al.*, 2016 ▸; Fitzpatrick *et al.*, 2017 ▸; Gremer *et al.*, 2017 ▸). Electron tomography is the visualization technique of choice for more complex samples, including the cellular environment. Owing to the poor signal-to-noise ratio (SNR), individual tomograms suffer from substantial noise artifacts. In the case where tomograms contain identical molecular units, they can be averaged by orientationally aligning particle volumes (Briggs, 2013 ▸). Recently, with the increase in data quality and improved image-processing routines, this approach also yielded near-atomic resolution maps from the HIV capsid (Schur *et al.*, 2016 ▸).

Regardless of whether they originate from single-particle analysis or subtomogram averaging, the resulting reconstructions are inherently limited in resolution and suffer from contrast loss at high resolution (Rosenthal & Henderson, 2003 ▸). In the raw reconstructions, the high-resolution features are barely visible as the amplitudes follow an exponential decay described by the *B*-factor quantity that combines the effects of radiation damage, imperfect detectors, computational inaccuracies and molecular flexibility. The Fourier shell correlation (FSC) is the accepted procedure to estimate resolution (Saxton & Baumeister, 1982 ▸; van Heel *et al.*, 1982 ▸; Rosenthal & Henderson, 2003 ▸) and can be compared with a given spectral signal-to-noise ratio (SSNR; Penczek, 2002 ▸). Consequently, *B*-factor compensation by sharpening is essential and is common practice. Sharpening is combined with signal-to-noise weighting to limit the enhancement of noise features (Rosenthal & Henderson, 2003 ▸). Based on sharpened maps, atomic models are built and are further improved by real-space or Fourier-space coordinate refinement (Adams *et al.*, 2010 ▸; Murshudov, 2016 ▸). This process is particularly challenging at the resolutions between 3 and 5 Å that are commonly achieved in cryo-EM. Recently, we proposed a method to sharpen maps by using local radial amplitude profiles computed from refined atomic models (Jakobi *et al.*, 2017 ▸). This method facilitates the interpretation of densities with resolution variation, but also requires the prior knowledge of a starting atomic model with correctly refined atomic *B* factors. Despite this advance, a more general approach is needed at the initial stages of density interpretation, in particular in the absence of prior model information. Tracing of amino acids derived from the primary structure as well as the placement of nonprotein components into density maps remains a laborious and time-consuming task. In particular, the EM density contains a large dynamic range of gray values for which only a small percentage of voxels are relevant for interpretation using isosurface-rendered thresholded representations. In practice, the process of choosing a threshold is helped by the empirical recognition of binary density features matching those of expected protein features at the given resolution. Therefore, it would be desirable to have more robust density-thresholding methods at hand to reduce subjectivity and provide statistical guidance in deciding which map features are considered to be significant with respect to background noise.

Extracting significant information from noisy data is a common problem in many fields of science. The simplest approach is based on thresholding corresponding to multiples of a standard deviation σ from an expected mean value. The experimental values are only considered to be significant when above this threshold and are rejected as noise when below this threshold. In X-ray crystallography and cryo-EM, this σ approach is commonly applied to the determined maps and σ thresholds are often reported when isosurface renderings of the density are displayed. In EM maps in particular, the σ levels reported for interpretation are not universal and will be chosen by the interpreter, as they vary from structure to structure between 1σ and 5σ and often to a smaller extent within the structure. The reason for the observed variation is that the high-resolution amplitudes of density peaks are very weak and can be compromised by noise after sharpening. In statistical theory, it has been recognized that the simple σ method tends to increase the probability of declaring significance erroneously with larger numbers of tests (Miller *et al.*, 2001 ▸), which is referred to as the multiple testing problem. To account for this effect, the probability of correct detection could be increased by controlling the false discovery rate (FDR; Benjamini & Hochberg, 1995 ▸). This statistical procedure has been applied to noisy images in astronomy (Miller *et al.*, 2001 ▸) and to time recordings of brain magnetic resonance images (Genovese *et al.*, 2002 ▸) to better discriminate signal from noise.

Owing to the low SNRs of cryo-EM maps at high resolution, separating signal from noise remains a daunting task. At present, the visualization and interpretation of the density requires experience of the operator and thus relies on subjectively chosen isosurface thresholds. As sharpening procedures also amplify noise alongside the high-resolution signal, a more robust assessment of the statistical significance of these features by a particular detection error is desirable. Here, we propose to apply the statistical framework of multiple hypothesis testing by controlling the FDR of cryo-EM maps. The resulting maps, which we refer to as confidence maps, represent the FDR on a per-voxel basis and allow the separation of signal from noise background. Confidence maps provide complementary information to EM densities from single-particle reconstructions and subtomogram averaging as they allow the detection of particularly weak features based on statistical significance measures.

## 2. Methods   

### 2.1. Statistical framework   

**Figure 1:**
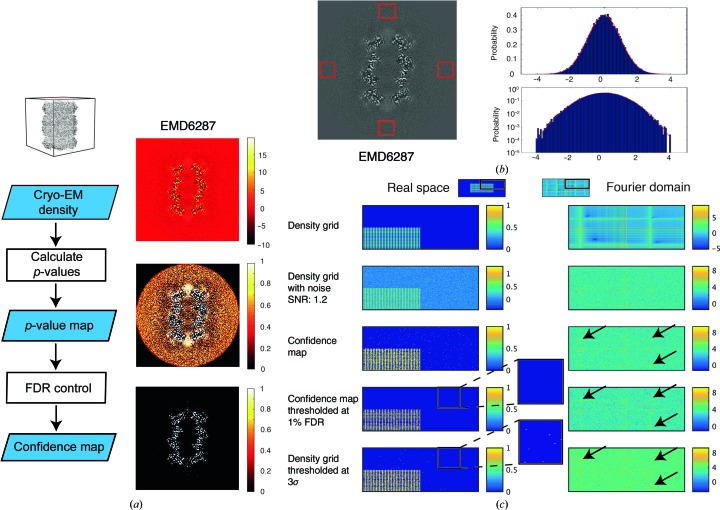
False discovery rate (FDR) analysis of cryo-EM maps. (*a*) Left: flowchart of confidence-map generation. The cryo-EM map is converted to *p*-values and finally FDR-controlled. Right: slice views through a cryo-EM map of 20S proteasome (EMD-6287) depicted at the respective stages of the algorithm (blue boxes) on the left. Note the strong increase in contrast when the sharpened map is converted to the confidence map. (*b*) Left: estimation of the background noise from windows (red) outside the particle. Right: histograms (top, probability on a linear scale; bottom, probability on a log scale) of the background window together with the probability density function of the estimated Gaussian distribution. (*c*) Evaluation of the algorithm on a simulated two-dimensional density grid. The upper right quadrant of images in real space (left column) together with the corresponding power spectrum in the Fourier domain (right column) are displayed. A density grid with added normally distributed noise at a signal-to-noise ratio of 1.2 leads to a loss of contrast at high resolution. Confidence maps recapitulate these high-resolution features (arrows), showing that high-resolution signal is detected with high sensitivity. FDR thresholding at 1% recovers a similar binary grid in comparison with 3σ thresholding while minimizing noise contributions and minimizing detected noise (enlarged insets).

In order to overcome limitations in interpreting density features with respect to significance, we applied multiple hypothesis testing using FDR control to cryo-EM maps. In this workflow, we estimate the noise distribution from the background of a sharpened cryo-EM map, apply subsequent statistical hypothesis testing for each voxel and control the FDR (Fig. 1 ▸ *a*). For the background noise, we assume a Gaussian distribution or, if required, an empirical density distribution where the mean and variance of the noise are estimated from four independent density cubes outside the particle density along the central *x*, *y* and *z* axes (Fig. 1 ▸ *b*). Subsequently, these estimates are used to obtain upper bounds to assess signal from the particle with respect to background noise (see Appendix *A* [App appa]). In addition, we assume that the cryo-EM density to be interpreted consists of positive signal (see Section 3). Therefore, statistical hypothesis tests are carried out by one-sided testing. To account for the total number of voxels and the dependency between voxels, *p*-values are further corrected by means of an FDR control procedure according to Benjamini & Yekutieli (2001 ▸), which has been designed to control the FDR under arbitrary dependencies. The FDR-adjusted *p*-values (or *q*-values) of each voxel are directly interpretable as the maximum fraction of voxels that have been mistakenly assigned to signal over the background.

As the *q*-values of the respective voxels provide a well established detection measure, we further explored their use for density presentation and thresholding. Based on the FDR, we inverted the map values to the positive predictive value (PPV) by PPV = 1 − FDR. When the map is thresholded at a PPV of 0.99, at least 99% of the binarized voxels are truly positive density signal within the map, corresponding to an FDR of 1%. We term these maps confidence maps, referring to the fact that PPVs serve as a measure of the confidence with which we can discriminate the signal from the noise. These confidence maps can then be visualized in the same way as usual cryo-EM maps with common visualization software, the difference being that the threshold for visualization is now given by 1 − FDR rather than the density potential.

### 2.2. Simulations   

The simulated images were 400 × 400 pixels in size. The scaled grid was generated by adding two orthogonal two-dimensional cosine waves with a period of five pixels, where all values smaller than 0 were set to 0, and multiplying the sum by a factor of 0.5 in order to scale the maximum to 1. The scaled grid was 200 × 200 in size and was embedded in the center of the 400 × 400 image. Gaussian-distributed noise with a mean of 0 and a given variance of 0.01 (Fig. 1 ▸ *c*), 0.1 (Supplementary Fig. S1*a*) or 1.33 (Supplementary Fig. S1*b*), respectively, was added to the grid image. The mean and variance for the multiple testing procedure were estimated outside the scaled grid and the FDR procedure was carried out as described. Simulations were implemented in *MATLAB* (MathWorks).

### 2.3. Software   

The algorithm is implemented in Python, based on NumPy (Walt *et al.*, 2011 ▸) and the mrcfile I/O library (Burnley *et al.*, 2017 ▸). Local resolutions were calculated using *ResMap* (Kucukelbir *et al.*, 2014 ▸). The software is available at https://git.embl.de/mbeckers/FDRthresholding. Figures were prepared with *UCSF Chimera* (Pettersen *et al.*, 2004 ▸).

## 3. Results   

### 3.1. FDR-based hypothesis testing yields improved signal detection in simulations   

In order to evaluate the principal performance of the proposed method on simulated data, we prepared a two-dimensional grid of continuous density waves (Fig. 1 ▸ *c*, left). We added white noise to a series of test images containing SNRs of between 3.9 and 0.3 as occur in the high-resolution shells of three-dimensional reconstructions when the FSC curve decreases from 0.67 to 0.143, often reported as the resolution cutoff. Firstly, we generated a test image with an SNR of 1.2 and noted that signal from high-resolution features cannot be detected in the power spectrum computed from the simulated noise images, although it is present in the noise-free power spectrum (Fig. 1 ▸ *c*, right). The detection of these high-resolution features, however, can be recovered from the corresponding confidence images that were generated as described above, even at SNRs ranging between 3.9 and 0.3 (Supplementary Figs. S1*a* and S1*b*). When comparing images thresholded at conventional 3.0σ levels with confidence images thresholded at a PPV of 0.99 or an FDR of 0.01 (referred to in the following as 1% FDR), we note that FDR-controlled thresholding allows more faithful detection of weak density features closer to noise levels. In this way, the density transformation to confidence images minimizes false-positive detection of pixels and improves the peak precision as adjacent noise peaks are suppressed (Supplementary Fig. S2).

### 3.2. Confidence maps from near-atomic resolution maps separate signal from background suitable for molecular structure interpretation   

**Figure 2:**
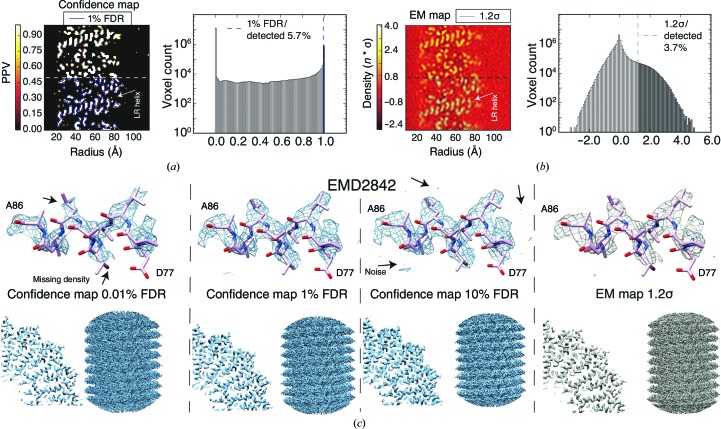
Confidence maps separate signal from noise for molecular-density interpretation. (*a*) Left: confidence map with a longitudinal section through the TMV coat protein displayed, indicating the α-helical pitch of the LR helix. The lower half shows the chosen contour at 1% FDR in blue with 5.7% of voxels detected. Right, the corresponding histogram of the confidence map with signal separated above 0.99 PPV (1% FDR). (*b*) Left: the same section as in (*a*) from cryo-EM density and the recommended threshold contoured at 1.2σ in gray with 3.7% of voxels detected. Right: the corresponding histogram of the cryo-EM density with thresholded values displayed in gray. (*c*) Isosurface-rendered thresholded confidence maps at 0.01%, 1% and 10% FDR (left, center left and center right, respectively) shown in blue and sharpened cryo-EM density with a 1.2σ threshold (right) in gray from TMV (EMD-2842). Shown are helix Ala86–Asp77 (top), a quarter cross-section (bottom left) and a side view (bottom right) of the TMV map.

In order to assess the potential of confidence maps for the interpretation of cryo-EM densities, we applied the algorithm to the near-atomic resolution map of *Tobacco mosaic virus* (TMV) determined at a resolution of 3.35 Å (EMD-2842; Fromm *et al.*, 2015 ▸). Variances could be estimated reliably outside the helical rod for a range of different window sizes from 10 to 30 voxels using the cryo-EM density (Supplementary Fig. S3). To generate the confidence map, we transformed the cryo-EM density to *p*-values and subsequently to confidence maps in an equivalent manner to the simulated confidence images above. Next, we inspected a longitudinal TMV section through the four-helical bundle of the coat protein and compared the confidence map with the cryo-EM density (Figs. 2 ▸ *a* and 2 ▸ *b*). The confidence map revealed backbone traces that contain values close to 1 corresponding to the helical pitch of the LR helix. They clearly stand out with respect to background noise, which is suppressed towards values of 0. The associated histogram of the confidence map revealed a strong peak beyond 0.99 PPV or below 1% FDR, separating signal from background and thresholding 5.7% of voxels within the density. In the case of the deposited cryo-EM map, the subjectively fine-tuned and recommended 1.2σ threshold also yielded a recognizable outline of helical pitch contours while detecting only 3.7% of voxels from the density. In analogy to isosurface-rendered cryo-EM densities, the confidence map exhibits recognizable structural details, such as the α-helical pitch and many side chains of the central helices (Fig. 2 ▸ *c*). When applying a lower FDR of 0.01%, the polypeptide density becomes discontinuous and smaller density features disappear. When using higher FDR thresholds such as 10%, noise starts to be included in the density. At the recommended 1% FDR threshold, the appearance of noise is minimal and well controlled in the confidence maps. This is in contrast to cryo-EM densities, where the appearance of noise is very sensitive to small changes in the threshold level, in particular at lower σ. In fact, the recommended 1.2σ contour includes only 52% of the atoms of the model, whereas the 1% FDR threshold contour already contains 73% of atoms with minimized noise. In order to include the same amount of atoms in a contour, a threshold of 0.7σ would be required, which will at the same time lead to a noticeable increase in obstructing noise. Furthermore, we also examined two additional confidence maps from EMDB model challenge targets determined at near-atomic resolution: 20S proteasome (Campbell *et al.*, 2015 ▸) and γ-secretase (Bai *et al.*, 2015 ▸) (Supplementary Figs. S4*a* and S4*b*). These confidence maps confirm the previous observation that when displayed at FDR levels of 1% they provide structural details at near-atomic resolution while effectively separating signal from noise.

### 3.3. Confidence maps provide a map-detection error with respect to background noise   

When confidence maps are generated from cryo-EM densities, we determine a voxel-based confidence measure of molecular density signal with respect to background noise. In principle, the confidence measure could also be interpreted as a broader error estimate of the EM map referring to the rate of falsely discovered voxels. However, the error that arises from a cryo-EM experiment is a comprehensive quantity which results from multiple contributions in the form of the solvent scattering and detector noise, as well as computational sources from alignment and reconstruction algorithms in addition to variation of the signal by multiple molecular conformations and radiation-damage effects (Frank & Liu, 1995 ▸; Penczek *et al.*, 2006 ▸). Estimating the complete series of error contributions to signal variation is currently not possible in the context of common cryo-EM collection schemes. In order to separate signal from background, however, it is sufficient to consider background noise from the solvent area. Rather than exact quantification of the experimental error, we aim to detect those voxels where the deviation is large enough to declare them statistically significant. Owing to the binary separation of signal from background, protein density variations are flattened in confidence maps. This property of confidence maps is particularly beneficial in the recognition of weak density features with intensities close to the background noise (see Section 3.5). Therefore, the most straightforward way of estimating noise is to measure the variance of the map solvent area. This variance mainly captures errors that arise from detector noise and solvent scattering, while neglecting the contributions of computation and local molecular variations. Detector noise can be considered to be distributed uniformly over the three-dimensional reconstruction, whereas the solvent-scattering distribution will not be uniform as the pure solvent noise next to the particle will be higher when compared with solvent noise projected through the particle owing to solvent displacement and variations of water thickness in the particle view (Penczek, 2010 ▸). Consequently, measuring noise in the solvent area of cryo-EM maps will lead to an effective overestimation of the background noise and therefore to an underestimation of the confidence (see Proposition 1 in Appendix *A* [App appa]). Although these deviations from a uniform Gaussian noise model do not allow absolute error determination, in practice an estimation of solvent variance can be used as a conservative upper bound for error rates without including errors arising from computation and molecular variation. Uncertainty from overfitting noise during the iterative refinement procedure is neglected in confidence maps and can therefore lead to underestimated FDRs. However, we do not consider noise overfitting to be a major problem with mature refinement algorithms (regularization of the likelihood) and the stable methods for initial model generation that have emerged in recent years. In conclusion, the error that arises from confidence maps should be considered to be a map-detection error with respect to background noise that deviates systematically from the absolute experimental error of the map intensities. Yet, the quantity remains beneficial in the process of interpreting cryo-EM densities.

### 3.4. Robustness of FDR-controlled density transformation   

In order to test the robustness of the approach, we systematically assessed the effects of the required input on the resulting confidence map. Firstly, we tested the influence of severely underestimating noise in confidence-map generation by using half or three quarters of the determined variance of the 20S proteasome densities (Supplementary Fig. S4*c*). The resulting confidence maps displayed at 1% FDR revealed an excessive declaration of background as signal, which poses a principal risk of overinterpretation. This principal risk, however, is less relevant when the variance measurements outside the particle proposed here are used, as we tend to overestimate noise (see above and Proposition 1 in Appendix *A* [App appa]). Therefore, we tested the effect of overestimating the variance by 1.25-fold, twofold and eightfold and generated confidence maps according to the defined procedure. The resulting confidence maps show the disappearance of map features at the 1% FDR threshold only when the variance is severely overestimated by a factor of 8; for small overestimations the effect is hardly noticeable in the appearance of the map. Another important noise-related parameter prior to the proposed procedure is the level of sharpening applied. Therefore, we applied a series of *B* factors from 0 to −250 Å^2^ to the 20S proteasome maps and converted them to confidence maps. Firstly, with increasingly negative *B* factors the corresponding confidence maps displayed at 1% FDR showed a loss of features owing to the decrease in relative significance. This is in contrast to cryo-EM densities, which become severely over-sharpened and the density features are dominated by noise (Supplementary Fig. S4*d*). Secondly, when under-sharpened maps are used for noise estimation, the maps will contain only low-resolution features lacking high-resolution detail at the respective significance level, in analogy to cryo-EM densities. Therefore, when over-sharpened maps are used for noise estimation, confidence maps inherently avoid the enhancement of noise features that could be mistakenly interpreted as signal. Although noise estimation is important for the procedure, tests show that smaller variance overestimation does not have a noticeable effect on the map interpretation of 1% FDR confidence maps. In conclusion, confidence maps represent a conservative way of displaying maps at defined significance while avoiding the problem of over-sharpening, which represents a principal benefit over the visualization of σ-thresholded sharpened EM densities.

### 3.5. Confidence maps facilitate the detection of weak density features   

**Figure 3:**
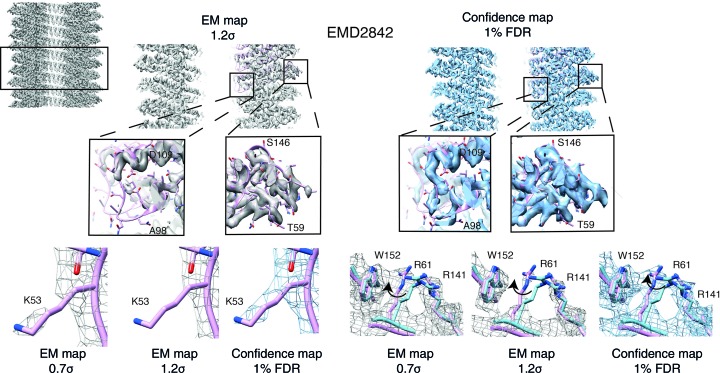
Confidence maps facilitate the detection of weak density features. Detailed comparison of TMV density and the corresponding confidence map. A slice view through the TMV rod with enlarged insets for inner and outer radii density (top). Lys53 side-chain density (left) and the molecular environment of Arg61 side chains (right) are shown at 0.7σ and 1.2σ thresholds and in a 1% FDR confidence map.

In order to evaluate further molecular details of the confidence map, we inspected more ambiguous density features of the TMV map. Peripheral density at lower and higher radii of the virus was notoriously difficult to interpret in previous work (Fromm *et al.*, 2015 ▸; Sachse *et al.*, 2007 ▸; Namba & Stubbs, 1986 ▸). For these regions, we found that well defined features are present in the 1% FDR confidence maps. The densities of the coat protein for the loops Gln97–Thr103 located at the inner radius and Thr153–Gly155 at the outer radius are not present in the respective EM map, but are clearly traceable in the 1% FDR confidence map (Fig. 3 ▸, center). In addition, side-chain density for Lys53 contacting the adjacent subunit was found to be clearly significant, while being discontinuous in the original map (Fig. 3 ▸, bottom left). Based on confidence maps, the re­adjustment of side-chain rotamers was possible, as illustrated for example by significant density for Arg61, which suggests a realignment of Arg61 to form stabilizing inter­actions with the aromatic Trp152 (Fig. 3 ▸, bottom right). The presented examples of TMV illustrate that confidence maps represent an alternative for density display, which can help in the process of molecular-feature detection. Although threshold adjustments in cryo-EM maps can also help model interpretation in ambiguous regions and enhance weak density features, they also amplify noise features and increase the risk of noise fitting.

We also tested the utility of the FDR-thresholding approach for conformationally heterogeneous densities and for three-dimensional classes of the V-ATPase–SidK complex (EMD-8724), which were determined at 6.8 Å resolution (Zhao *et al.*, 2017 ▸). Firstly, the deposited EM map contains very weak EM density for the bacterial effector SidK owing to low occupancy and flexible motion. The corresponding confidence map of the V-ATPase–SidK complex reveals that the SidK density is not significant as continuous density when thresholded at 1% FDR as it is too noisy for further analysis (Supplementary Fig. S5*a*). In Section 3.7, we will deal with cases of local resolution and SNR variation that can be accommodated by a locally adjusted FDR procedure. Secondly, we analyzed confidence maps from three conformational states generated by three-dimensional classification (EMD-8724, EMD-8725 and EMD-8726). The generated confidence maps thresholded at 1% FDR of states 1, 2 and 3 confirm previous observations about the rotational states of SidK using EM maps (Supplementary Fig. S5*b*) and show that computationally separated three-dimensional classes can be equally well visualized using this approach. Taken together, confidence maps provide an inherent significance level associated with the density and minimize false-positive noise detection. In this way, confidence maps can guide atomic model interpretation of cryo-EM density maps, in particular in density regions of ambiguous quality.

### 3.6. Confidence maps from subtomogram averages   

**Figure 4:**
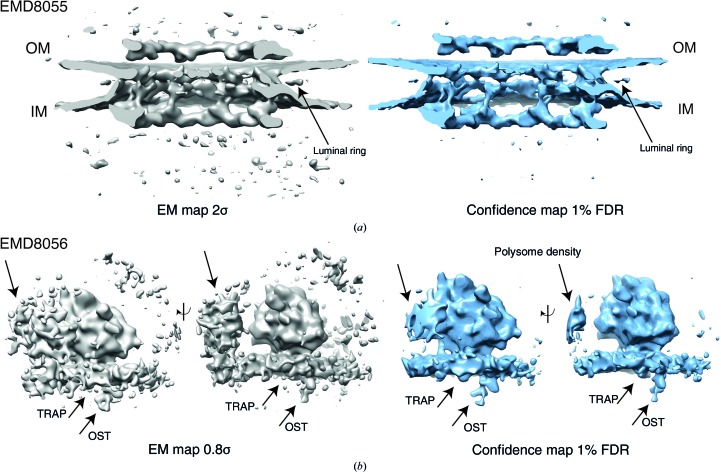
Confidence maps from subtomogram averages. (*a*) Nuclear pore structure at 90 Å (EMD-8055) from eight pore particles: cryo-EM map at 2.0σ threshold (left, gray) and confidence map at 1% FDR threshold (right, blue). Note that the confidence map minimizes the appearance of noise. (*b*) ER-associated ribosome structure at 35 Å resolution (EMD-8056) in two side views at a 0.8σ threshold (left) and 1% FDR confidence map (right). Note that in confidence maps weaker densities assigned to the peripheral protein complexes TRAP and OST (arrows) can easily be visualized in the absence of noise.

We further explored whether structures determined at lower resolution may also benefit from this approach. For this purpose, we examined the *in situ*-determined subtomogram average of the HeLa nuclear pore complex computed from eight pore particles at 90 Å resolution (Mahamid *et al.*, 2016 ▸). The deposited map clearly shows continuous densities for the cytoplasmatic and inner ring molecules, whereas density below and above the pore is noisy when visualized at a threshold of 2.0σ (Fig. 4 ▸ *a*). The corresponding 1% FDR confidence map shows continuous features for the ring structure with minim­ized noise, which makes interpretation straightforward. In order to generate a confidence map for a subtomogram average structure, care must be taken to identify areas of noise devoid of any signal in order to estimate the noise variance reliably (Supplementary Fig. S6*a*). The same tomograms recorded from lamella of HeLa cells also yielded a subtomogram average of ER-associated ribosomes. The ribosome structure itself could be determined at 35 Å at the membrane, with the weak density below the membrane ascribed to a translocon-associated protein complex and an oligosaccharyltransferase (Mahamid *et al.*, 2016 ▸). The corresponding densities can only be visualized at low thresholds corresponding to 0.8σ, while increasing the amount of background noise and hampering molecular interpretation (Fig. 4 ▸ *b*). The 1% FDR confidence maps, however, display the additional protein complexes in the absence of noise. In this case, the confidence map discriminates between specific association of the TRAP complex and the looser association of ribosomes within the polysome assembly. Further, we examined the deposited and confidence maps of the 23 Å resolution nuclear pore structure determined by subtomogram averaging (Appen *et al.*, 2015 ▸; Supplementary Fig. S6*d*). While the overall densities look very similar, we focused our comparison on the ambiguous density assignment of the linker region of Nup133. The presence of density in the 1% FDR confidence maps confirms the continuity of this density stretch and the author’s interpretation of placing the Nup133 linker region connecting the N-terminal β-propeller and C-terminal α-helical domain (Supplementary Fig. S6*d*, upper right). In addition, we identified additional densities in the connecting densities between the inner and nuclear ring as well as between the inner and the cytoplasmic ring (Supplementary Fig. S6*d*, bottom). Both densities are not visible at the recommended threshold of 2.1σ, but they are reliably displayed in the 1% FDR confidence map. In contrast to clearly defined features in high-resolution protein structures (for example secondary structure or side chains), we generally do not know what the density features of such subtomogram averages should look like, which makes manual thresholding as well as the validation of additional densities difficult. Taken together, confidence maps generated from lower resolution subtomogram averages assist in the density interpretation by separating the signal with respect to background noise.

### 3.7. Confidence maps benefit from local SNR adjustment in cases of resolution variation   

**Figure 5:**
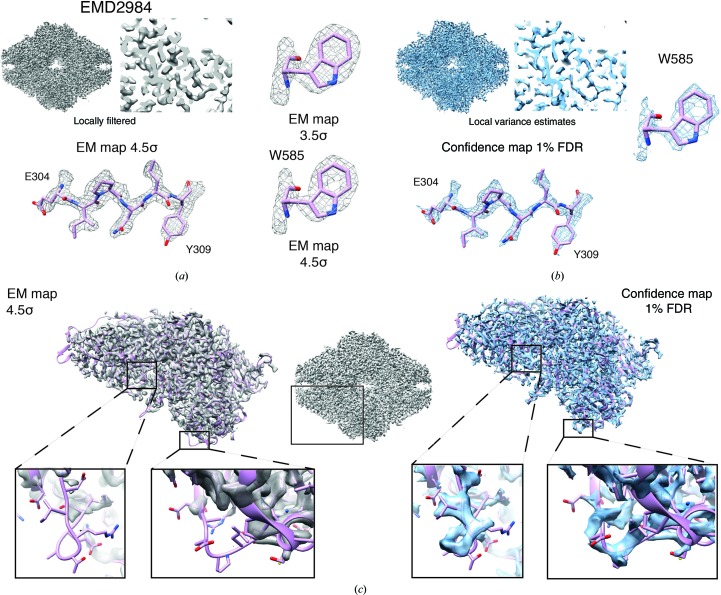
Confidence maps benefit from local SNR adjustment based on local resolution. (*a*) Locally filtered β-galactosidase (EMD-2984) cryo-EM map (gray) displayed at a 4.5σ threshold (left) and (*b*) confidence map (blue) including signal-to-noise adjustment based on local resolution at a 1% FDR threshold (right) in side view and cross-section. High-resolution features such as Glu304–Glu398 and holes in the aromatic rings of Trp585 in the 3.5/4.5σ-thresholded cryo-EM map (*a*) in comparison with the 1% FDR confidence map (*b*). (*c*) Comparison of density features from peripheral loop regions not covered by density in the locally filtered cryo-EM map (left) compared with the 1% FDR confidence map that shows densities for the respective loops.

After establishing their usefulness for maps covering a range of resolutions, we wanted to further explore how FDR-controlled confidence maps cope with large resolution differences within a single map. For this purpose, we analyzed the very high-resolution map (2.2 Å resolution) of β-galactosidase (β-gal; EMD-2984; Bartesaghi *et al.*, 2015 ▸) in more detail as it covers resolution ranges from 2.1 to 3.8 Å. In order to reveal high-resolution details in the center of the map high sharpening levels were required, and consequently less well resolved parts in the periphery of the map resulted in over-sharpened densities. When we applied our method to the cryo-EM density volume, we found the 1% FDR confidence to be well defined in the center of the map but to fade out for large parts of the periphery, supporting the *B*-factor test series using the 20S proteasome (Supplementary Fig. S4*d*). We reasoned that when the resolution differs across the map as a consequence of molecular flexibility and computational errors, the SNR will vary in correspondence. To compensate for these effects, noise levels can be adjusted in cryo-EM maps by applying local low-pass filtrations in Fourier space according to local resolutions (Cardone *et al.*, 2013 ▸). Consequently, a local variance can be estimated for each voxel by applying the same low-pass filter to the background noise windows (Supplementary Fig. S7*a*). Application of this procedure followed by FDR control yields a more evenly distributed 1% FDR confidence map including the β-gal periphery (Figs. 5 ▸ *a* and 5 ▸ *b*, top). At the same time, side-chain details such as holes in aromatic rings can be resolved at the same significance level, as exemplified for Trp585, in analogy to the appropriately filtered density (Figs. 5 ▸ *a* and 5 ▸ *b*, bottom). Closer inspection of the cryo-EM density shows that we did not observe density for the peripheral loops of the β-gal complex at the 4.5σ threshold but clearly detected continuous loop density at an FDR of 1% in the resolution-compensated confidence map (Fig. 5 ▸ *c*, left and right). These observations show that the statistical power of the procedure can be improved, *i.e.* the amount of missed signal can be reduced, while still controlling the FDR by the incorporation of local resolution information (see Appendix *A* [App appa] for a detailed discussion).

We recently introduced a local map-sharpening tool for cryo-EM maps based on refined atomic *B* factors (Jakobi *et al.*, 2017 ▸). When refined atomic coordinates are available, the concept of resolution-compensated confidence maps based on adjusted variances derived from local resolution filtering can easily be extended by scaling the radial amplitude falloff of the noise window against the local reference model for estimating the resulting local noise levels (Supplementary Fig. S7*b*). In order to directly compare confidence maps generated by different filtering or scaling approaches, we focused on inspection of the peripheral regions of the β-gal enzyme as the densities are weak, in particular for loops extending from the particle. When we compared the confidence map of this region generated using the local resolution filtering with the original confidence map, we confirmed the observation that adjustments according to local resolutions improve the density connectivity (Supplementary Figs. S10*a* and S10*b*). When we used the local amplitude scaling approach, we obtained a confidence map with improved density coverage when compared with the original confidence map but less coverage when using local resolution filtering (Supplementary Figs. S10*b* and S10*c*). In combination, when local variance is estimated based on local amplitude scaling and filtering, we find optimal coverage of the density and the atomic model (Supplementary Fig. S10*d*). Another example from the EMDB model challenge is the TRPV1 channel determined at 3.4 Å resolution (EMD-5578; Liao *et al.*, 2013 ▸). The structure contains a well defined transmembrane region and a more flexible cytoplasmic domain that is less well resolved. The application of locally adjusted SNRs to the confidence map yields a map with well interpretable density including molecular details (Supplementary Figs. S7*c* and S7*d*). In analogy to the examples above, the cytoplasmic domain is only visible at lower thresholds than the core of the protein. The 1% FDR confidence map captures all density occupied by the protein, including the more flexible regions in the cytoplasmic domain. The example of the TRPV1 channel confirms the observation for β-gal that local resolution differences need to be taken into account for the correct generation of confidence maps. When maps exhibit a strong local variation of noise owing to molecular flexibility and computational errors, local variances can be estimated based on local resolution measurements or on local sharpening procedures and yield well interpretable confidence maps at a single FDR threshold.

### 3.8. Confidence maps confirm the detection of bound molecules   

**Figure 6:**
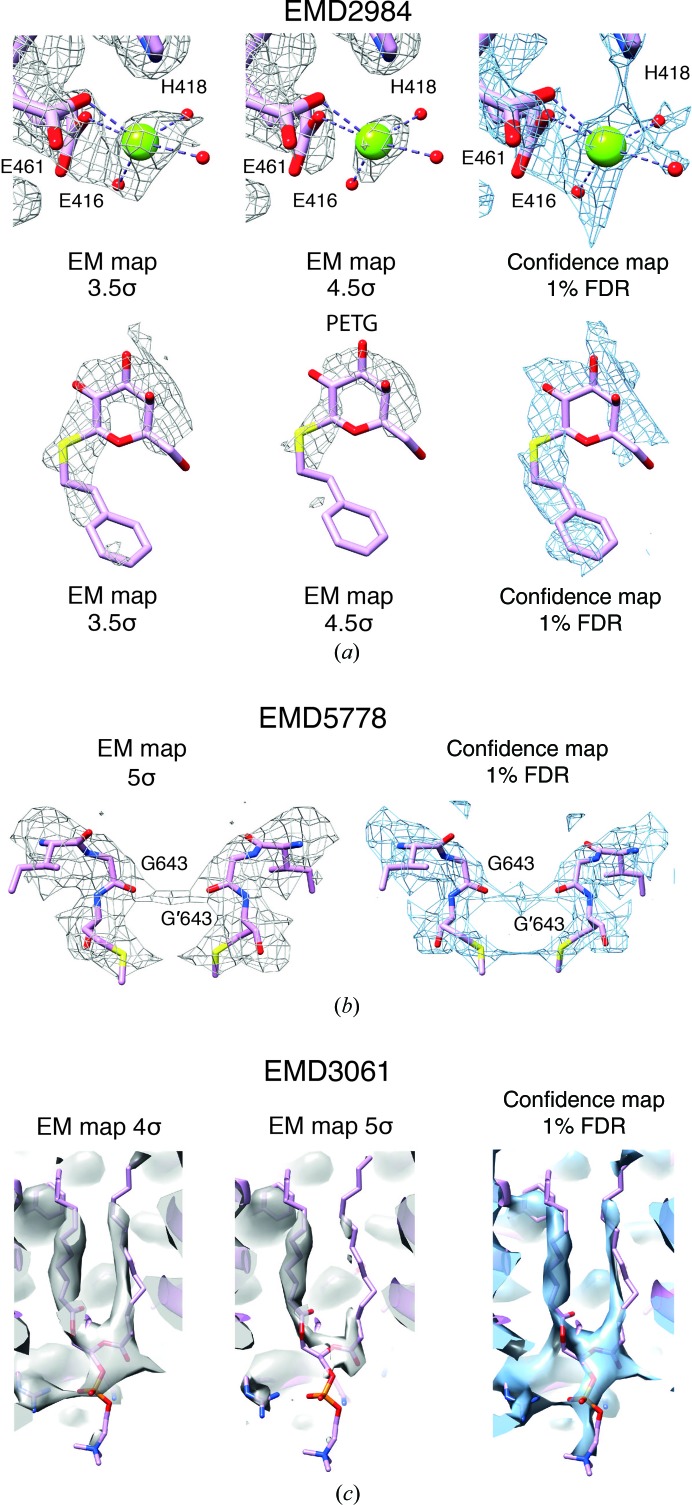
Confidence maps confirm the localization of nonprotein components. (*a*) β-Galactosidase (EMD-2984) with 3.5/4.5σ-thresholded cryo-EM maps (left and center, gray) and a 1% FDR-thresholded confidence map (right, blue). Top: the Mg^2+^ ion is coordinated by Glu461, Glu416, His418 and three H_2_O molecules. Bottom: density of bound PETG ligand in 3.5/4.5σ-thresholded cryo-EM maps and the 1% FDR confidence map. (*b*) TRPV1 channel (EMD-5778) with a 5σ-thresholded cryo-EM map (left) and a 1% FDR-thresholded confidence map (right): the selectivity filter formed by the carbonyls of symmetry-related Gly643 residues. The presence of a putative ion is supported by the confidence map. (*c*) γ-Secretase (EMD-3061) with 4σ- and 5σ-thresholded cryo-EM maps (left) and a 1% FDR-thresholded confidence map (right). The confidence map reveals density for both acyl chains of phosphatidylcholine at a single threshold.

The majority of near-atomic resolution maps obtained by cryo-EM are in the resolution range between 3 and 4.5 Å. Although main-chain and large side-chain densities can often be modeled reliably, smaller side chains and ordered non­protein components such as water molecules and ions are inherently difficult to model at these resolutions and pose the risk of noise fitting. Therefore, we investigated whether confidence maps can help to mitigate this problem and inspected a putative Mg^2+^ site coordinated by Glu416, Glu461, His418 and three additional H_2_O molecules inside the β-gal enzyme. We rigidly placed the Mg^2+^ ion and coordinated water molecules based on the 1.6 Å resolution X-ray crystal structure (Wheatley *et al.*, 2015 ▸; PDB entry 4ttg) and superposed them onto the deposited EM density map. The map at the lower 3.5σ threshold shows convincing density for only two of the three water molecules (Fig. 6 ▸ *a*, top left). In contrast, the 1% FDR confidence map based on local variance estimation reveals distinct density peaks for all three suspected H_2_O molecules (Fig. 6 ▸ *a*, top right). Furthermore, β-gal had been imaged in the presence of the small-molecule inhibitor PETG. Location and conformational modeling of the ligand remains challenging owing to flexibility and lower occupancy (Fig. 6 ▸ *a*, bottom left). Ligand placement is facilitated using confidence maps, with density being well resolved for the complete small-molecule inhibitor (Fig. 6 ▸ *a*, bottom right). The confidence density confirms the previous re-refinement of the inhibitor position and conformation (Jakobi *et al.*, 2017 ▸). In addition, we also tested whether the detection of smaller ions can be facilitated by confidence maps. For this purpose, we turned again to the TRPV1 channel and inspected the density surrounding Gly643, known as the selectivity filter for the ions passing the channel. The deposited map reveals a density peak in the symmetry center that is compatible with a small ion. In support, the confidence map also shows a density peak at the same position, supporting the presence of an ion with a confidence of 1% FDR (Fig. 6 ▸ *b*, bottom right). In correspondence, there are multiple cryo-EM structures reporting putative ion densities along an array of carbonyls forming an inner cavity of the channel (Lee & MacKinnon, 2017 ▸; McGoldrick *et al.*, 2018 ▸). Closer inspection of the γ-secretase complex reveals significant density for a membrane-embedded phosphatidylcholine (PC) lipid molecule. In order to detect the two PC acyl chains, the deposited EM map requires thresholding at two different σ levels of 4 and 5, presumably owimg to differences in chain mobility (Fig. 6 ▸ *c*). In contrast, the corresponding 1% FDR confidence map encompasses most of the density of the two acyl chains without the need for threshold adjustments. In conclusion, confidence maps from cryo-EM structures possess minimized noise and can be directly used to evaluate the significance of density features that are present by providing a map-detection error that, for example, 1% of the peaks are expected to be falsely discovered. Using complementary information for the interpretation of cryo-EM structures will help to reduce the subjectivity involved in the process of density interpretation.

## 4. Discussion   

In the current manuscript, we introduced FDR-based statistical thresholding of cryo-EM densities as a complementary tool for map interpretation. This approach has been used successfully in other fields of image-processing sciences (Genovese *et al.*, 2002 ▸). Based on a total of five near-atomic resolution EM maps from the EMDB model challenge (http://challenges.emdatabank.org), one intermediate resolution (6.8 Å) structure and three subtomogram averages in the resolution range 90–23 Å, we showed that the use of 1% FDR confidence maps is well suited for detailed molecular-feature detection and results in better confidence, in particular for the assignment of weak structural features. Although different σ levels ranging between 1 and 5 could be used for the interpretation of relevant cryo-EM map features for all maps, confidence maps thresholded at a common 1% FDR level show a consistent interpretability of molecular features for these maps. The advantage of confidence maps is that they effectively separate signal from a background noise estimate by assigning a confidence scale from 0 to 1 and at 1% FDR. In this way, they show a consistent inclusion of signal while minimizing noise. In contrast, for cryo-EM densities small changes of the isosurface σ threshold can have severe consequences for the interpretability of molecular features and bear the risk of mistakenly including noise. Therefore, confidence maps and associated FDR thresholds provide a common and conservative thresholding criterion for the interpretation of cryo-EM maps.

Included in the algorithm is a direct assessment of the signal significance with respect to background noise associated with particular density features visible in cryo-EM maps, which adds an additional objectivity to the reporting of ambiguous density features. Based on these properties, high-resolution confidence maps will be helpful in initial atomic model building when no or few atomic reference structures are available and for the assessment of critical details such as side-chain conformations and nonprotein molecules in the density. The use of these maps will improve the quality of initial atomic models before launching real-space or reciprocal-space atomic coordinate refinement (Murshudov, 2016 ▸; Adams *et al.*, 2010 ▸), which should proceed with sharpened or alternatively model-based sharpened maps as refinement targets (Jakobi *et al.*, 2017 ▸). Molecular interpretation based on confidence maps is not limited to maps of close-to-atomic resolution, as we have demonstrated its benefit for cases of intermediate-resolution single-particle and subtomogram averaging with three maps ranging in resolution from 7 to 90 Å. In these cases, the interpretation of an unassigned density using a confidence level is a beneficial property, in particular in the absence of atomic model information.

We also showed that the generation of confidence maps is a robust procedure. From the sharpened cryo-EM density, we compute the CDF from the solvent background, which in most cases can be approximated by a Gaussian distribution. In addition, we assume protein density to be positive, as the overwhelming majority of density for determined atoms resides in positive density. Moreover, we find that the region selected for noise estimation is critical as it has to contain pure noise devoid of signal. We found this particularly important for generating confidence maps from subtomogram averages with particle boundaries that are less well defined. Generally, when estimating background noise outside the particle we tend to overestimate noise owing to a lower ice thickness in the particle regions. Smaller deviations from noise estimation show little effect on the conversion to confidence maps (Supplementary Fig. S4*b*). We show that when suboptimally sharpened input maps are used to generate confidence maps, the operator avoids the common risk of mistakenly interpreting noise as signal in over-sharpened cryo-EM densities. In contrast, confidence maps generated from over-sharpened input maps will only result in an insufficient declaration of the density signal, which is an important safety feature. Once noise has been estimated, the procedure of generating confidence maps is statistically clearly defined (Benjamini & Hochberg, 1995 ▸; Benjamini & Yekutieli, 2001 ▸) and does not contain any free parameters to optimize. Only in cases of substantial resolution variation owing to molecular flexibility and computational errors may it be required to locally adjust SNRs by including prior information through local resolution filtering. More sophisticated approaches such as amplitude scaling can also be used in cases where atomic reference structures are available. Adjusting FDR control based on prior information is routinely implemented in other applications of statistical hypothesis testing (Chong *et al.*, 2015 ▸; Ploner *et al.*, 2006 ▸). With this manuscript, we provide a program that requires a three-dimensional volume as input and allows specification of the location of the density windows used for noise estimation. The presented implementation including local resolution filtration is computationally fast, taking from 30 s to 2 min on a Xeon Intel CPU for the maps produced in this manuscript.

We presented several cases in our simulation and EMDB maps where confidence maps displayed weak structural features more clearly while minimizing the occurrence of false-positive pixels (Figs. 1–6). This is a particularly useful property of confidence maps. Weak densities close to inherent noise levels are present in most cryo-EM maps and they result as a consequence of the molecular specimen as well as from the applied computational procedures. For example, they can originate from side-chain mobility in the form of multiple rotamers or side-chain-specific radiation damage (Fromm *et al.*, 2015 ▸; Allegretti *et al.*, 2014 ▸; Bartesaghi *et al.*, 2014 ▸). In addition, ligands, including small organic compounds or larger protein complex components, may have lower occupancy or partial flexibility (Zhao *et al.*, 2017 ▸). In many complexes, peripheral loops exposed to the solvent tend to have larger molecular flexibility than the core of the protein (Hoffmann *et al.*, 2015 ▸). We showed that thresholding confidence maps yields higher voxel-detection rates than thresholding in common cryo-EM densities. We believe that is a result of the fact that the human operator prefers to recommend a more conservative σ threshold to avoid the excessive inclusion of noise, while as a consequence one misses out on signal. Using confidence maps, this type of noise can be suppressed and as a result more reliable signal can be interpreted.

With the increasing number of near-atomic resolution cryo-EM structures, the process of building atomic models has become increasingly important, but remains time-consuming and labor-intense. Confidence maps can assist the user throughout this process. In X-ray crystallography, multiple complementary maps are used routinely in the process of model building. Real-space model building and optimization is typically performed using maximum-likelihood-weighted 2*mF*_o_ − *DF*_c_ maps, assisted by *mF*_o_ − *DF*_c_ difference maps to highlight errors in the model. Various forms of OMIT maps computed from phases of models in which a selection of atoms (for example a ligand) has been omitted are used to confirm the presence of ligands and ambiguous density. Similarly, confidence maps display a complementary aspect of cryo-EM maps in helping to reduce ambiguity in density interpretation of, for example, weakly bound ligands, alternative side-chain rotamers and conformationally heterogeneous structures, including incomplete or flexible parts of the complex. It is evident that confidence maps would not be suitable for model refinement, as they do not discriminate the scattering masses of different atoms or the relative uncertainties of atomic positions. These properties are usually modeled by atomic electron form factors and atomic displacement factors (atomic *B* factors). However, owing to the increased precision of density peaks and noise suppression, it is perceivable that confidence maps could be used to guide positional coordinate refinement if implemented as a peak-searching procedure. In addition, defined confidence values for density stretches should also be useful and potentially beneficial for automated model-building approaches. Interpreting cryo-EM densities by means of an atomic model is often the final step of a cryo-EM experiment. In practice, atomic models can even be used as a validation tool to examine density features for side chains at expected positions. One of the key advantages of the confidence maps proposed here is that they can be generated without prior knowledge of an atomic model. As the conversion of cryo-EM densities to FDR controlled maps is conceptually simple and computationally straightforward, confidence maps could be routinely consulted to provide complementary information of statistical significance during the intricate process of interpreting ambiguous densities in cryo-EM structures resulting from molecular flexibility or partial occupancy.

## Supplementary Material

Supplementary Figures.. DOI: 10.1107/S2052252518014434/pw5002sup1.pdf

## Appendix A

### A1. Statistical model

For each voxel in the reconstructed three-dimensional volume, where the voxels are indexed *i*, *j*, *k*, the intensity *X*_*i*,*j*,*k*_ is modeled as

where ∊_*i*,*j*,*k*_ is a real-valued random variable representing the background noise with mean μ_0,*i*,*j*,*k*_ ∈  and variance σ^2^_*i*,*j*,*k*_ ∈ , and where μ_*i*,*j*,*k*_ ∈  is the true intensity as observed without background noise.

We developed an algorithm by means of multiple hypothesis testing, which controls the maximum amount of false-positive signal in the map, *i.e.* the FDR with respect to background noise. Firstly, we limit the tested voxels to the reconstruction sphere, and voxels located outside a diameter larger than the box size are disregarded as they arise from a smaller subset of averaged images than the voxels inside. Secondly, we focus on the detection of voxels with positive deviations from background noise (see Section A3). In addition, voxels that contain significant signal are affected by further sources of noise such as flexibility, incomplete binding of ligands and structural heterogeneity, leading to intensity variations of the signal. Consequently, these sources lead to an increase of the variance for these voxels as part of the incoherent signal, which we do not consider here as it is going beyond the scope of detecting signal beyond background. Background noise of experimental cryo-EM data, however, poses principal challenges to the statistician, as it can result in non-uniform distributions across the map: although background noise variances from images of uniform noise over the pixels can be assumed to be uniform over the central sphere (Supplementary Fig. S8*c* right), background noise outside the particle is higher when compared with background noise affecting the particle itself owing to solvent displacement and variations of the relative ice thickness at the particle (Penczek *et al.*, 2006 ▸). Therefore, estimating noise in the solvent region outside the particle could lead to an overestimation of the actual influence of the background noise on the particle (see Section 3.3). Although this may cause several problems for comprehensive probabilistic modeling, these estimates can be interpreted as conservative bounds for the signal significance of the particle over background noise. For this reason, we use multiple hypothesis testing in order to calculate these upper bounds for detection errors of false-positive rates, as we prove in Proposition 1. In cases when alternative noise estimates are available, they can be supplied as additional input to the procedure in order to generate confidence maps.

For each voxel a *z*-test is carried out, which identifies significant deviations from background noise. The value of the test statistic *Z* at each voxel is then given as

where *x*_*i*,*j*,*k*_ ∈  is the reconstructed mean intensity at the respective voxel. We are testing for true intensity μ_*i*,*j*,*k*_ higher than 0; thus, the null and alternative hypotheses for each voxel become

The null hypothesis *H*_0_ states that the true intensity μ_*i*,*j*,*k*_ at the respective voxel is 0, *i.e.* no signal beyond background noise, while the second hypothesis *H*_1_ states the deviation towards higher values. Testing for deviations towards negative values, *i.e.* negative densities, is easily accomplished in this setting by multiplying the normalized map intensities *z*_*i*,*j*,*k*_ by −1, leading to a left-sided test procedure. Both options can be chosen by the user.

Under the null hypothesis *H*_0_ and by approximating the background noise with a Gaussian distribution (Kucukelbir *et al.*, 2014 ▸; Vilas *et al.*, 2018 ▸), the test statistic *Z* follows a standard Gaussian distribution. The *p*-values in our procedure are then calculated as

where *Z*_*i*,*j*,*k*_ is a random variable representing the test statistic at voxel *i*,*j*,*k*, *z*_*i*,*j*,*k*_ is the particular realization and  is the background noise as estimated from the solvent area and the cumulative distribution function Φ() of the standard Gaussian distribution. Alternatively, *p*-values can also be calculated in a nonparametric way without any assumptions about the underlying background noise distribution by simply replacing the cumulative distribution function Φ() of the standard Gaussian distribution with the empirical cumulative distribution function  estimated from the sample of background noise, given as

This allows the complete procedure to be carried out without any distribution assumptions. However, comparisons show that the background noise can be well approximated with a Gaussian distribution even in the tail areas, which are most important for the calculation of *p*-values (see Section A3, Fig. 1 ▸ *b* and Supplementary Fig. S8*a*). The respective method for *p*-value calculation, *i.e.* nonparametric or with Gaussian assumption, can be chosen by the user. All cases presented in the manuscript, if not stated otherwise, were calculated with the assumption of Gaussian-distributed background noise. Note that the *p*-values defined here differ only marginally from the *p*-values commonly used for one-sided testing in a way that for all voxels with intensities smaller than the estimated mean noise level  their value is set to 1. This definition allows the control of the FDR in the more general setting of allowed overestimated mean and variance (see Proposition 1).

### A2. Multiple testing correction

The respective hypothesis tests are applied to each voxel in the three-dimensional volume. To account for the multiple testing problem with up to more than a million tests, we choose to control the FDR. Control in this context means giving upper bounds for the error that occurs. The FDR is defined as the expected amount of false rejections, *i.e.*

where  is the number of false rejections,  is the number of true rejections and  denotes the expectation value. Owing to dependencies between hypotheses at voxels close to each other, we choose the Benjamini–Yekutieli procedure (Benjamini & Yekutieli, 2001 ▸), giving an FDR-adjusted *p*-value for each voxel; these are often referred to as *q*-values. To describe the adjustment of *p*-values according to Benjamini and Yekutieli in more detail and for ease of notation, we will now use a sequence of voxels from the map and denote the number of hypotheses, *i.e.* tested voxels, by *m*. The *p*-values *p_i_*, *i* = 1, …, *m* are then sorted, from small to large, resulting in sorted *p*-values *p*_(*i*)_, *i* = 1, …, *m*. *q*-values are then calculated as

where *m* is the number of hypotheses, *k* is a running index and . By recognizing the correct index in the sequence of voxels for each index (*i*), *i* = 1, …, *m* in the sorted array and subsequent conversion into the three-dimensional volume, we can assign each voxel position *i*, *j*, *k* its corresponding *q*-value. In order to interpret the resulting map, the *q*-value for each voxel then gives the minimal FDR that has to be imposed at the thresholding in order to call the respective voxel a significant deviation from the background. The final value associated with voxel *i*, *j*, *k*, *q*′_*i*,*j*,*k*_, is then calculated as

where *q*_*i*,*j*,*k*_ is the *q*-value at the voxel indexed with *i*,*j*,*k*. Thus, visualization of the map at a value of 0.99 corresponds to a maximal FDR of 1%, or a minimal PPV of 99%, and therefore means that of all the visible voxels at this threshold, a maximum of 1% are expected to be background noise.

Next, we show that the presented procedure with *p*-values as defined above controls the FDR even in the case of overestimated background noise, *i.e.* by using the possibly overestimated background-noise estimates from the solvent area in (2) for all voxels.

*Proposition 1*. Consider Gaussian-distributed random variables representing the background noise at all voxels *i*, *j*, *k* in the three-dimensional map with true mean μ_0,*i*,*j*,*k*_ ∈  and variance . Moreover, let  and , ,  for all *i*, *j*, *k*, the overestimated background-noise parameters. Then, , where  corresponds to the *q*-value as defined in (7) and calculated with our procedure with parameters  and  corresponds to the *q*-value as obtained with the true parameters  and .

*Proof*. In order to prove the statement, we will now re­capitulate the algorithm and prove the inequality at all necessary steps. We start by showing that the true *p*-value at voxel position *i*, *j*, *k*, *p*_*i*,*j*,*k*_, is smaller when compared with the *p*-value  calculated from the overestimated background-noise parameters using (4). In other words, we want to show that  or, equivalent to this, . If  then the statement is trivial, because  and *p*_*i*,*j*,*k*_ ≤ 1, which is a general property of *p*-values.

For , considering (2) and (4), it follows that

As the error function erf() is monotonically increasing, it is sufficient to show that

Because  and thus also *x*_*i*,*j*,*k*_ − μ_0,*i*,*j*,*k*_ ≥ 0, as well as , we have

where in the last inequality it was used that  and . This gives the desired result of .

Recapitulating the calculation of *q*-values in (7) together with the conversion of the three-dimensional volume to a sequence, it follows that

where *m* is the number of hypotheses, *k* is a running index and . This gives the desired result:

□

As the Benjamini–Yekutieli procedure controls the FDR when using true parameters, our procedure (*i.e.* the Benjamini–Yekutieli procedure applied to the modified *p*-values) will give a more conservative estimate of the FDR (as shown in Proposition 1). Therefore, our algorithm controls the FDR sufficiently well by giving an upper conservative bound for the FDR. Thus, Proposition 1 states that even in the setting of non-uniform background noise with higher noise levels in the region of background-noise estimation, the FDR is controlled and thus robust in the sense that the maximum FDR is still guaranteed. Furthermore, it must be mentioned that estimates of the background-noise levels are not the only factor that contributes to FDR estimation. Both the number of voxels as well as their dependencies within the map have an important influence and are considered in the FDR adjustment. This makes the generation of confidence maps even with severely overestimated background-noise parameters a powerful procedure (Supplementary Fig. S4), where powerful is used here in its statistical sense of decreasing the error of missing true signal. However, the power of the procedure can be further increased, *i.e.* the amount of true missed signal reduced while controlling the FDR, by including information about local resolutions, the cutoffs in reciprocal space beyond which no signal is expected, while at the same time controlling the FDR.

### A3. Choice of positive-density model with Gaussian background noise

Although the model of Gaussian noise is often used to approximate background noise in cryo-EM images and maps (Sigworth, 1998 ▸; Scheres, 2012*a* ▸; Kucukelbir *et al.*, 2014 ▸; Vilas *et al.*, 2018 ▸), it is important to analyze actual maps to better understand deviations from this assumption. For this purpose, we analyzed a total of 32 deposited cryo-EM densities from 2 to 8 Å resolution and compared the empirical cumulative density function (CDF) with the ideal Gaussian CDF (Supplementary Fig. S8*a*). It is apparent that all of them follow the ideal Gaussian CDF closely. For each map, we assessed normality by Anderson–Darling hypothesis testing (Anderson & Darling, 1954 ▸) and found that 75% and 87.5% of the maps do not significantly deviate from normality when conservative thresholds corresponding to 1% and 0.1% family-wise error rates (FWER) are chosen (Supplementary Fig. S8*b*). One of the reasons for the observed deviations from an idealized Gaussian distribution is a result of the three-dimensional reconstruction procedure. In principle, when truly aligned images containing white Gaussian noise are combined by linear inversion, the obtained three-dimensional volume will also have a Gaussian distribution. In practice, in cases when uncertainties reside on the five orientation parameters, background noise is not necessarily Gaussian-distributed. Moreover, the resulting three-dimensional reconstructions will contain local correlations, *i.e.* ‘colored noise’. Therefore, we analyzed the resulting noise of three-dimensional reconstructions generated from pure noise images with even angular sampling. The resulting amplitude spectrum shows that it differs from pure white noise owing to correlations between adjacent pixels (Supplementary Fig. S8*c*, left). Furthermore, the variances estimated for each voxel from 900 reconstructions show that they can be approximated as uniform over the central sphere (Supplementary Fig. S8*c*, right).

For the map EMD-6287, which deviates strongly from normality according to the Anderson–Darling test, we generated a confidence map using the Gaussian and the empirical CDF. We inspected these confidence maps (Supplementary Fig. S8*d*) and found that the visual agreement between the two maps is very high. To highlight potential differences, we computed a difference map between the two confidence maps created by the two approaches and observed no systematic variation when deviation from normality was assumed. Therefore, when interpreting confidence maps, small deviations from normality do not appear to have practical limitations. In order to rule out any potential unforeseen effects when maps deviate more strongly, we routinely implemented monitoring of the degree of deviation from the ideal Gaussian CDF. For instance, when the deviation of the empirical CDF from the Gaussian CDF exceeds 0.01, referring to the fact that the *p*-values deviate by more than 1%, we can optionally use the empirical CDF for the generation of confidence maps.

The second assumption of the proposed confidence map is that the protein gives rise to positive density in cryo-EM maps. When inspecting EM density maps, it is evident that not all signal present in the map is positive, which might be important to consider for atomic coordinate refinement. Therefore, we analyzed whether significant negative densities can be detected in confidence maps generated from inverted densities. Indeed, the confidence maps from negative densities reveal significant signal in regions between protein density, often in a spatially complementary way (Supplementary Fig. S9*a*, left). Using the independently determined X-ray structure of the 20S proteasome (PDB entry 1pma; Lowe *et al.*, 1995 ▸), we tested whether negative density coincides with the atomic model. Overall, negative density has only a very small 2.5% overlap with atoms, which is close to the predicted false-discovery rate of 1% (Supplementary Fig. S9*b*). When using positive density, however, we find that a large fraction of 60% of the PDB atoms are found in the 1% FDR-contoured confidence map and that 10% of this volume is occupied by modeled atoms. In conclusion, we show that negative density presents significant signal in cryo-EM maps, but that only a very small fraction is occupied by atoms. The largest fraction of negative densities are found next to positive protein density, most likely owing to the fact that the molecular density is lower than in the particle-surrounding solvent area. Based on this analysis and our objective to identify those voxels that arise from protein density, we include the restraint of testing for positive signal into the generation of confidence maps and include an additional option to test for negative signal, which could be used for further investigation of negative densities.

### A4. Testing with local filtering

In the presence of extreme resolution variation, using uniformly sharpened and filtered maps will lead to confidence maps with insufficient representation of features in both areas with *B* factors either lower than the average or higher than the average. Therefore, in the next two sections, we will show how noise levels can be locally adjusted and subsequently estimated by the inclusion of local resolution information as well as atomic *B* factors and how this can be used to increase the power to detect weaker features while controlling the FDR. Local filtration of EM maps according to the local resolution (Cardone *et al.*, 2013 ▸) has been shown to be a powerful approach as it leads to local reductions in background noise. These variations of noise levels between different voxels at different resolutions from local filtering can be also accounted for in the generation of confidence maps. For each voxel, a map duplicate volume is filtered at the corresponding resolution and the noise distribution is estimated from the solvent area outside the particle. This procedure results in three three-dimensional maps, the estimates of local variances of the background noise at each voxel after local filtration, the estimates of local means of the background noise at each voxel after local filtration and the locally filtered map. These three maps are subsequently used for the testing procedure. Thus, the value of the test statistic (2) is calculated by

where *x*_*i*,*j*,*k*_ ∈  is the intensity of the locally filtered map at voxel *i*, *j*, *k*, and  and  are the local mean and standard deviation estimate of the background noise at the respective voxel. All subsequent steps of the algorithm remain identical, as well as the validity of Proposition 1.

### A5. Testing with local amplitude scaling

As for the local filtration, local amplitude scaling gives rise to varying noise levels at different voxels. In order to obtain both mean and variance estimates for each voxel after local amplitude scaling, a duplicate window outside the particle containing pure noise is scaled according to the rolling window used in local amplitude scaling for each voxel, *i.e.* the amplitudes of the Fourier transform of the box containing pure noise at frequency *s*, denoted as *F*_noise_(*s*), are multiplied by a frequency-dependent sharpening factor , which is consequently given as

where  and  are rotationally averaged amplitudes of the Fourier transform at frequency *s* given at the respective rolling window for the sharpened and the observed experimental map, respectively. The noise distribution is then estimated from the scaled noise sample. In analogy to the case of locally filtered maps, this procedure again results in three three-dimensional maps of estimated means, variances and intensities of the locally sharpened map for each voxel that can be incorporated with (13) in the testing procedure. Proposition 1 remains valid.
