# Supplementary material for: Thresholding of cryo-EM density maps by false discovery rate control
Source: IUCrJ. 2019 Jan 1;6(Pt 1):18–33. doi: 10.1107/S2052252518014434 (PMC6327189; doi:10.1107/S2052252518014434)
Supplement: Supplementary file 1 [file m-06-00018-sup1.pdf]

IUCrJ

Volume 6 (2019)

Supporting information for article:

Thresholding of cryo-EM density maps by false discovery rate control

Maximilian Beckers, Arjen J. Jakobi and Carsten Sachse

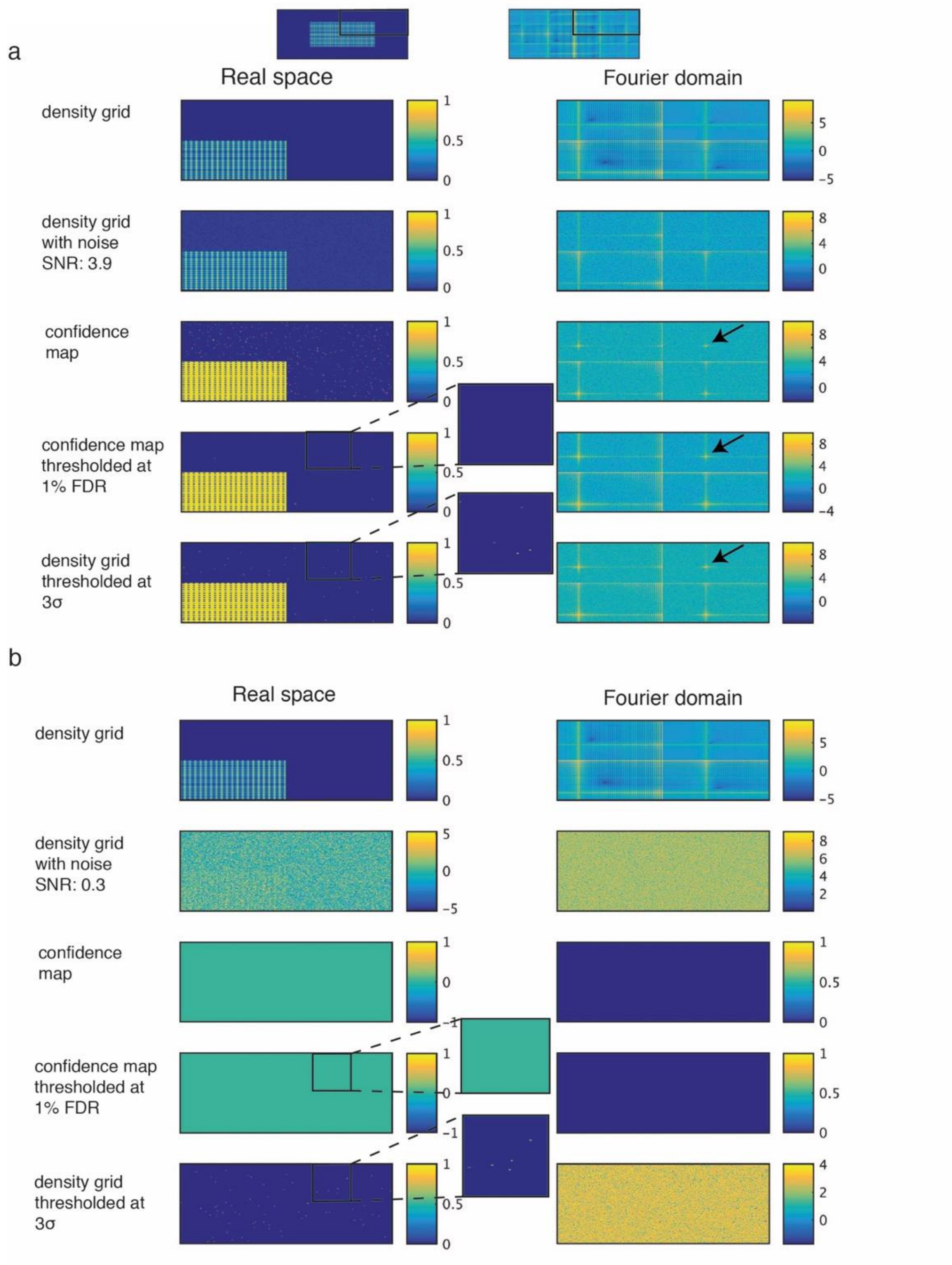

Figure S1 Comparison of σ and FDR thresholding of simulated density grids with varying signal-to-noise ratios. Thresholding with simulated density grids at signal-to-noise ratios and variance of (a) 3.9 (0.01) and (b) 0.3 (1.33), respectively. The same simulations as in Fig. 1c are repeated with lower and higher variances of the background noise. At low signal-to-noise ratio, the 1% FDR thresholding is devoid of false positives whereas conventional 3σ-thresholding approach yields many false positive pixels (zoomed inset).

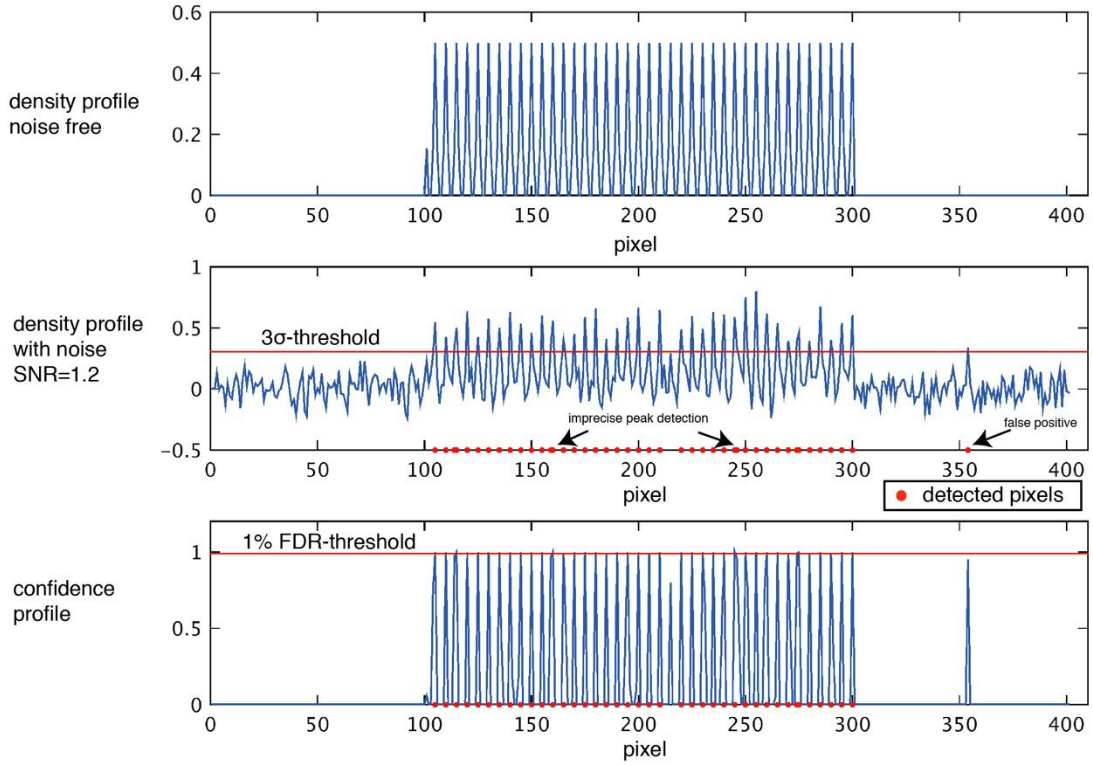

Figure S2 Effect of σ and FDR thresholding on 1D density profiles. One-dimensional stacked plots of grid density with noise-free original (top), at signal-to-noise ratio of 1.2 (center) and confidence map (bottom). The noisy density grid is thresholded at 3σ and the confidence map is thresholded at 1 % FDR. Conventional 3σ-thresholding yields higher rates of false positives and some imprecise peak positions (arrows).

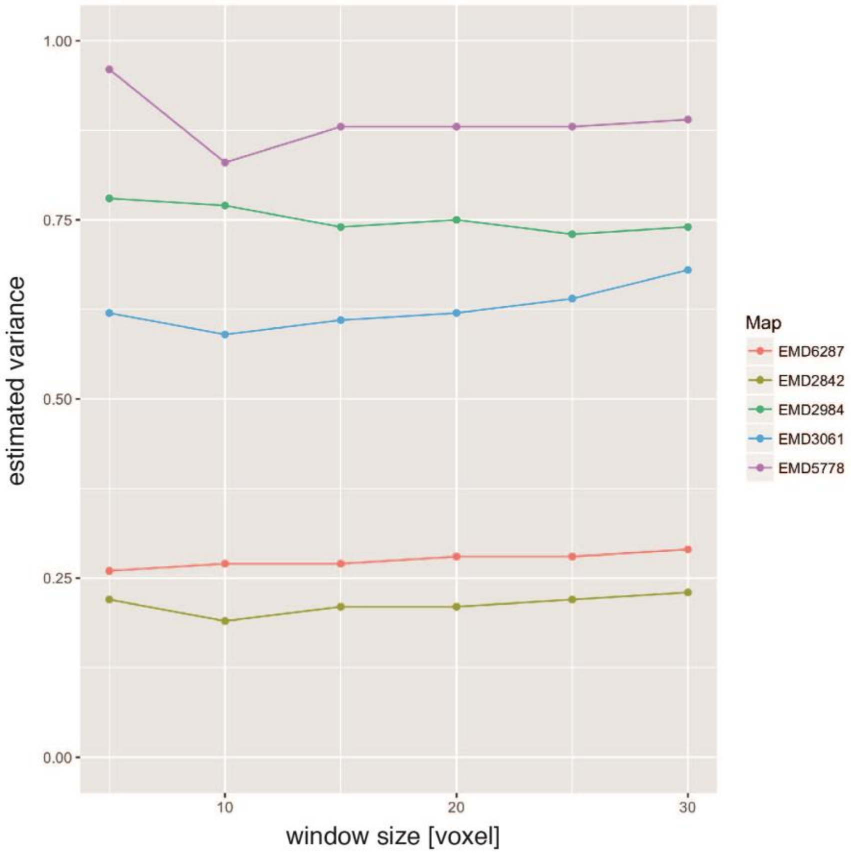

Figure S3 Effect of window size on estimated variance. Estimated variance is stable with increasing window size from 5 to 30 voxels for a series of EMD entries.

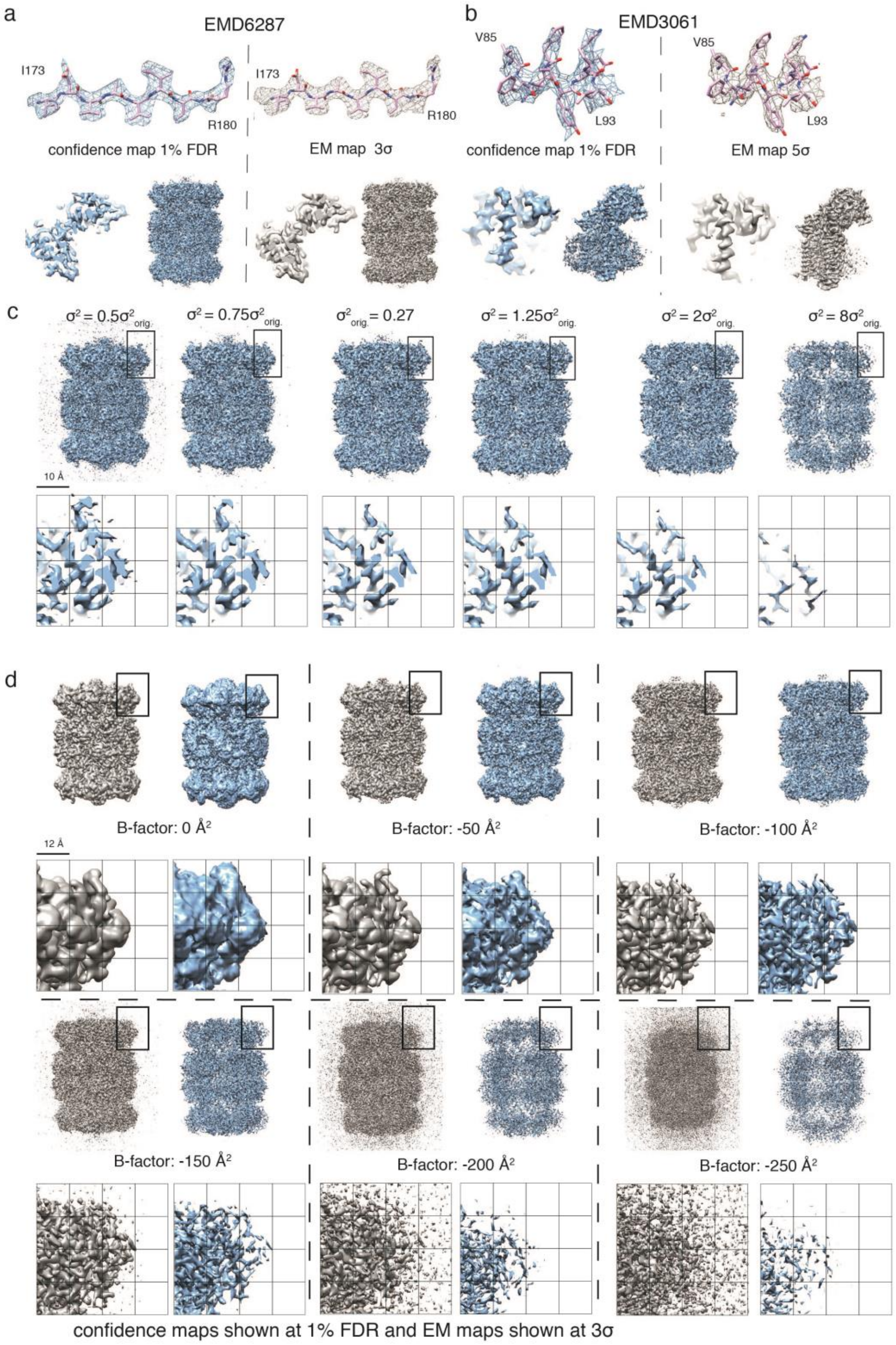

Figure S4 Confidence maps and effect of incorrect noise estimation. (a) 20S proteasome map (EMD 6287) comparison of 1% FDR density (left) and 3σ-thresholded map (right). Shown are molecular details from I173 – R180 (top), slice view (bottom left) and side view (bottom right) of density. (b) γ-secretase map (EMD 3061) comparison of 1% FDR confidence map and 5σ-thresholded map. (c) Six confidence maps of 20S proteasome (EMD-8267) including magnified inset based on

incorrect variance estimation: 1st and 2nd left noise is underestimated by 0.5 and 0.75 times the variance (σ^2). In comparison with the correctly estimated noise (3rd), they show excessive noise features declared as signal at 1 % FDR. When noise is overestimated, which is more likely for cryo-EM maps, confidence maps are quite insensitive to changes in map appearance. For multiples like 1.25σ^2 and 2σ^2 no apparent density changes become visible (4th and 5th) unless strong overestimation like 8σ^2 (6th) leads to disappearance of map features at a 1 % FDR threshold. (d) When applying a series of B-factors to the 3D reconstruction of the 20S proteasome map, we see that with higher B-factors, sharpened EM densities become dominated by noise whereas corresponding confidence maps displayed at 1 % FDR show disappearance of significant features thereby avoids over-interpreting noise features.

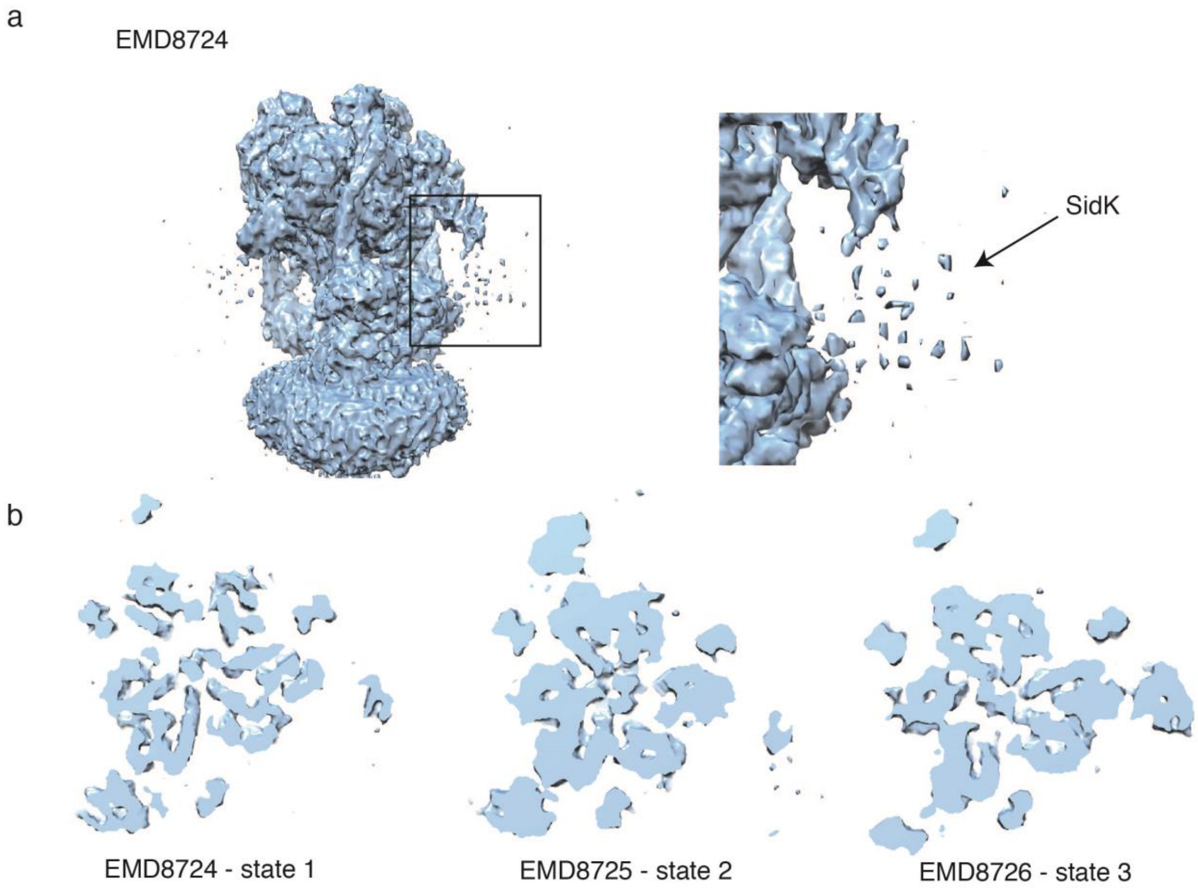

Figure S5 Confidence maps of compositionally and conformationally heterogeneous complexes. (a) Confidence map of yeast V-ATPase with Legionella pneumophila effector SidK (EMD8724) at 1% FDR (left) together with a zoom on the flexible domains of SidK (right). The confidence map shows significant density for the flexible domains, however, not as continuous density. (b) Slices through confidence maps of 3D classified cryo-EM maps. Different rotational states can be resolved in the confidence maps.

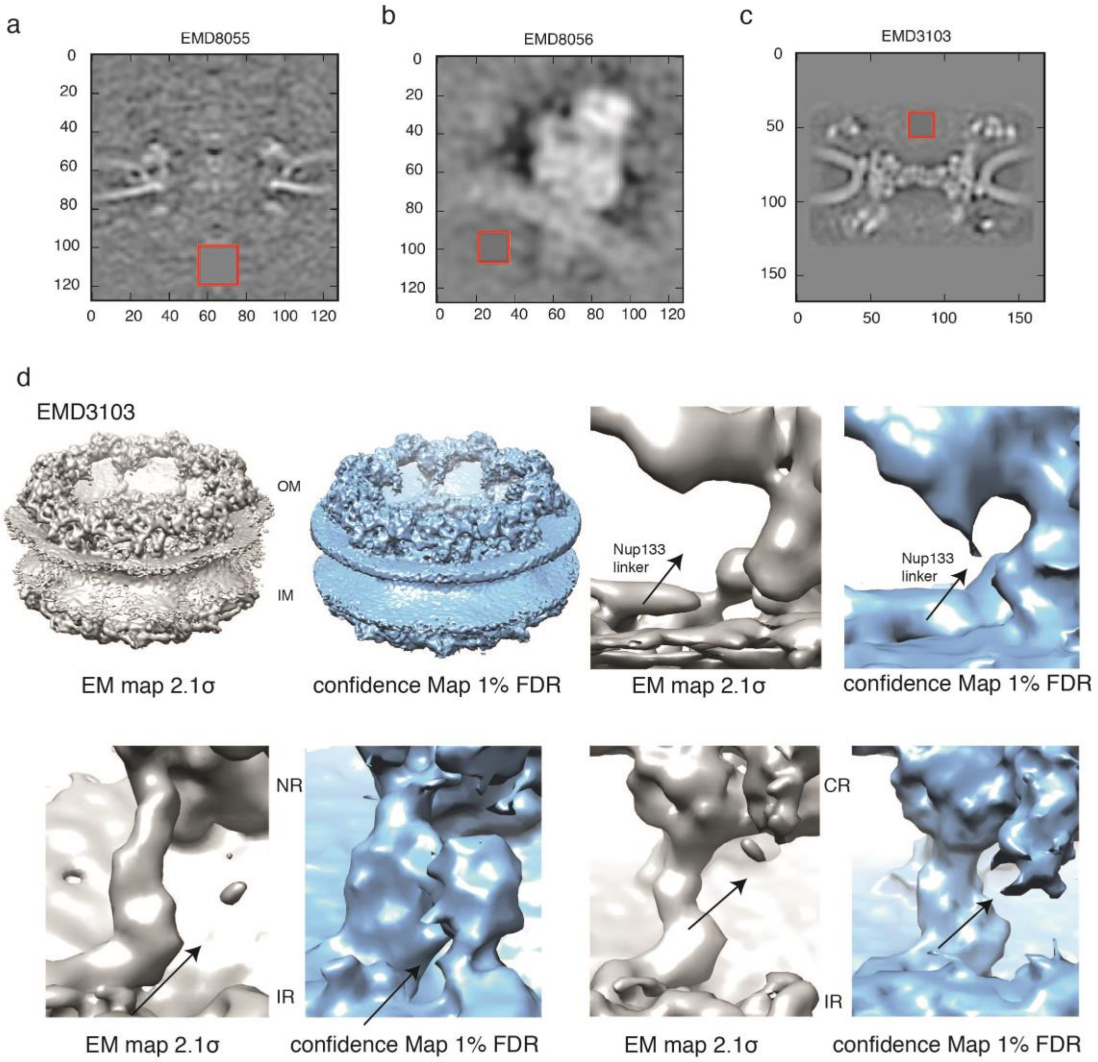

Figure S6 Noise estimation in subtomogram averages. Gray-scale density slices with red windows for the voxel region used for variance estimation: (a) EMD 8055: nuclear pore from HeLa cells by FIB-SEM, (b) EMD 8056: ER-associated ribosomes, (c) EMD3103: 23 Å resolution nuclear pore subtomogram average. (d) Nuclear pore structure at 23 Å resolution (EMD 3103) comparing cryo-EM map at 2.1σ threshold (left) and 1 % FDR confidence map (right). Comparison of map pairs for Nup133 linker density (top right), densities located between inner and nuclear ring (bottom left) and inner and cytoplasmic ring (bottom right). In contrast to sharpened cryo-EM maps at 2.1σ threshold, confidence maps show consistently densities at the connections between the inner and outer rings at 1 % FDR threshold (arrows).

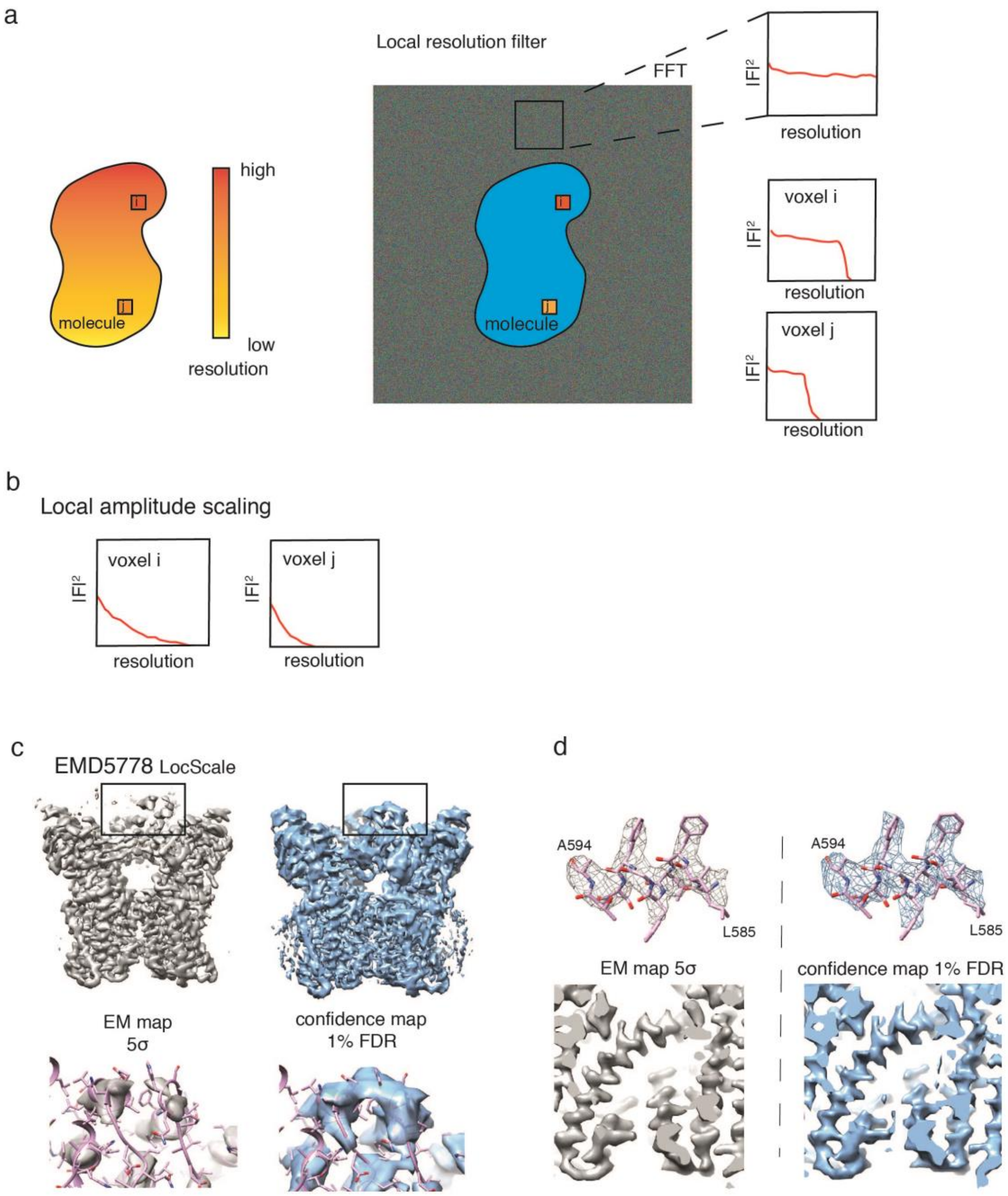

Figure S7 Variance adjustment based on local resolution and local amplitude profile. (a) Adjusting the local signal-to-noise ratio based on local resolution measurements: for each voxel, the background windows are filtered according to the local resolution at the respective voxels in order to estimate the noise levels of each voxel in the locally filtered map. (b) In analogy, local sharpening is applied to background noise in order to estimate resulting local noise distributions. (c) TRPV1 (EMD5778) side view (top) with zoom-in to peripheral cytoplasmic domain density (bottom) comparing LocScale density displayed at 5σ threshold (left) and 1 %FDR confidence map. (d) Detailed density stretch A594 – L585 (top) and transmembrane helix S5 including S4-S5 linker (bottom) comparing LocScale density and 1 % FDR confidence map.

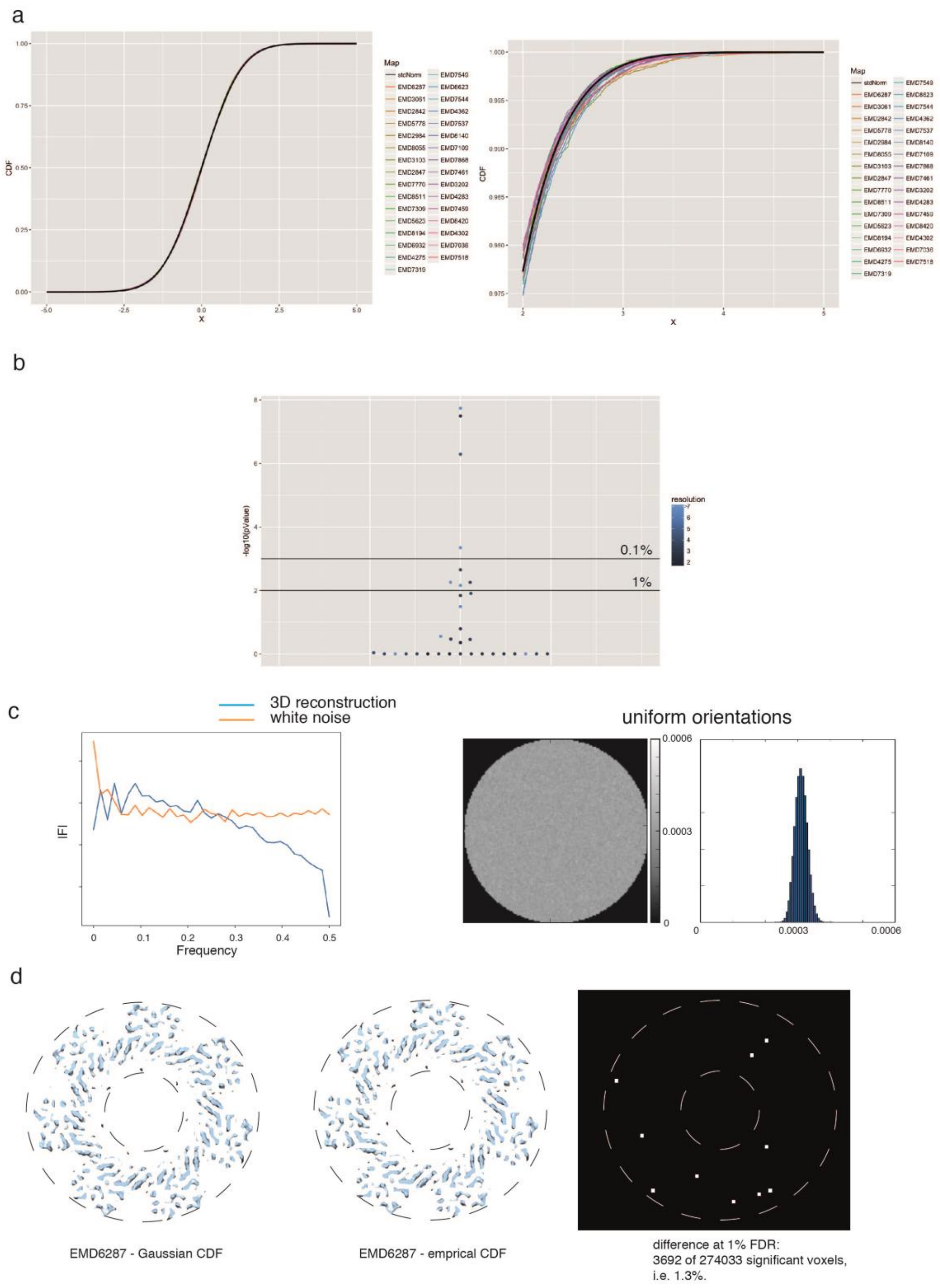

Figure S8 Analysis of normality of cryo-EM densities. (a) Left. Overlay of 32 cumulative density functions (CDF) derived from the above EMDB entries with ideal Gaussian CDF in black. Right. Zoomed inset to better highlight small differences. (b) 32 map entries are assessed with respect to normality according to the Anderson-Darling test, significance thresholds are displayed 1.0 and 0.1 % respectively. (c) Left. Rotational power spectrum of a 3D reconstruction of white noise images in

comparison with pure white noise spectrum. Right. Slice through 3D volume of variances estimated from 900 independent reconstructions from Gaussian white noise images with similar uniform orientations together with a histogram of the estimated variances, showing that background noise can be assumed uniform over the central sphere in the reconstructed volume. (d) Cross-sectional view of confidence maps generated of EMD6287 using Gaussian and empirical CDF. Difference map between 1 % FDR binarized confidence maps in the respective image slice.

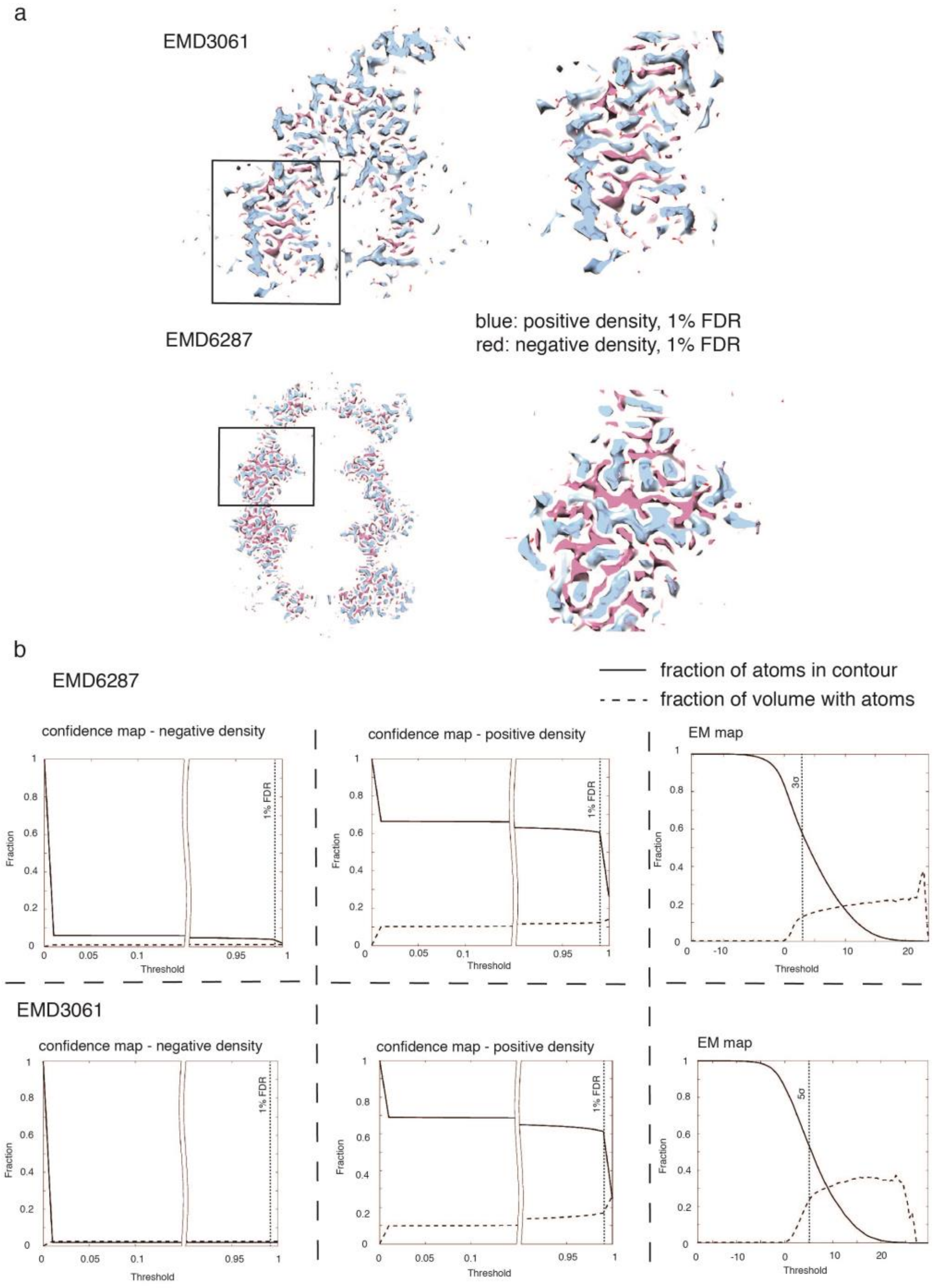

Figure S9 Analysis of positive and negative densities using confidence maps. (a) Overlay of 1% FDR positive (blue) and negative (red) confidence maps from original and inverted densities of

EMD3061 (top) and EMD6287 (bottom) respectively. (b) Comparison of detected signal with corresponding atomic models by determining the fraction of overlap of atoms with volume and fraction of volume with atoms as a function of threshold for negative (left), positive (center) confidence maps and cryo-EM maps (right), respectively.

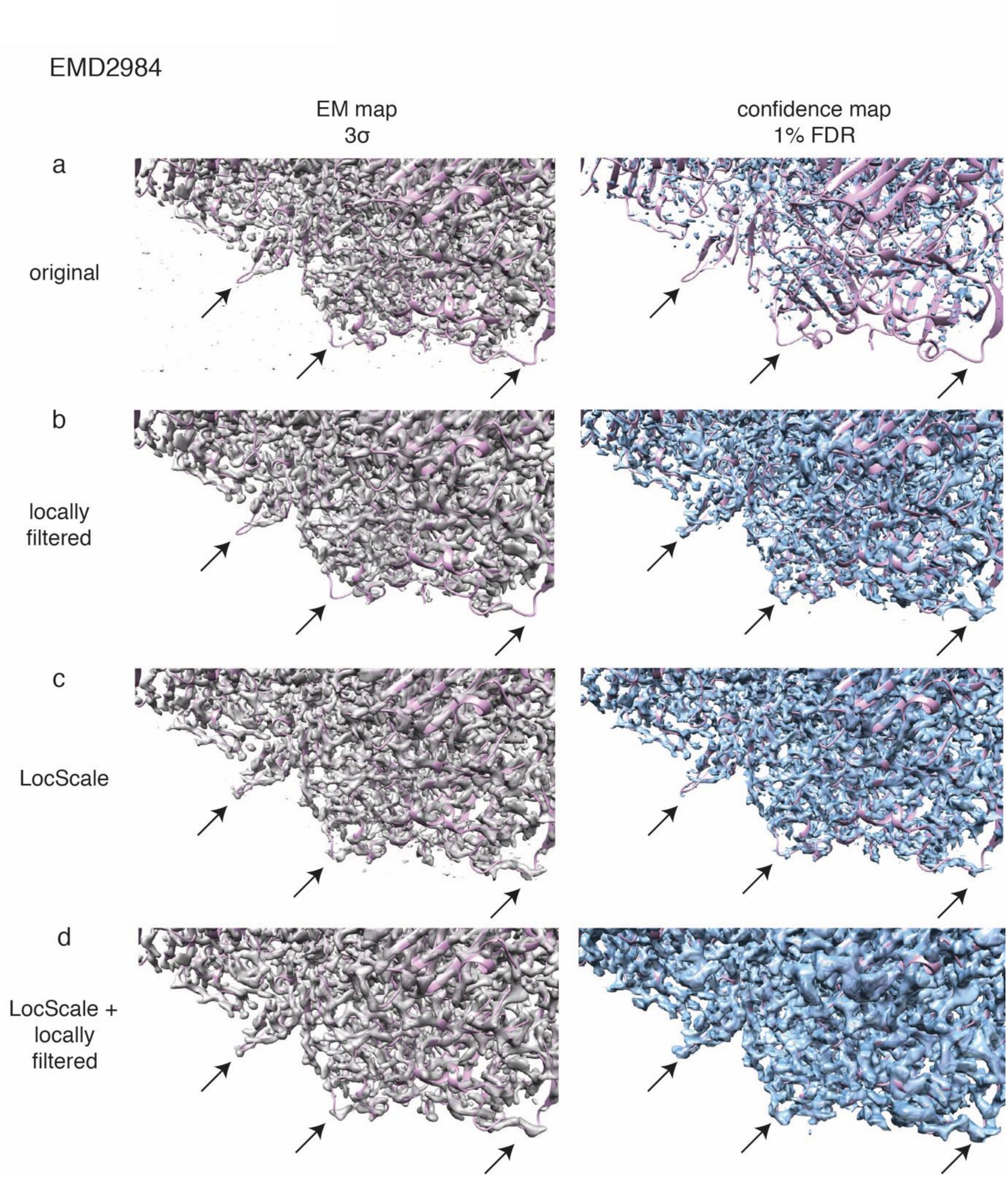

Figure S10 Effect of local variance adjustments on confidence maps. β-galactosidase (EMD 2984) cryo-EM map at 3.0σ threshold (left, gray) and 1 % FDR confidence map based on different post-

processing methods (right, blue). Global sharpening with uniform filtering, local filtering based on local resolution measurements, local sharpening and the combination of local sharpening with local filtering were compared. Confidence maps were generated with local noise estimate based on local resolution measurement, locally scaled window from a model reference structure and the combination of both, which in this case shows the best preservation of molecular density with respect to confidence.
